# Comparative analysis of small RNAs released by the filarial nematode *Litomosoides sigmodontis in vitro* and *in vivo*

**DOI:** 10.1371/journal.pntd.0007811

**Published:** 2019-11-26

**Authors:** Juan F. Quintana, Sujai Kumar, Alasdair Ivens, Franklin W. N. Chow, Anna M. Hoy, Alison Fulton, Paul Dickinson, Coralie Martin, Matthew Taylor, Simon A. Babayan, Amy H. Buck

**Affiliations:** 1 Institute of Immunology and Infection Research and Centre for Immunity, Infection & Evolution, School of Biological Sciences, University of Edinburgh, Edinburgh, United Kingdom; 2 Unite Molecules de Communication et Adaptation des Microorganismes (MCAM, UMR 7245), Sorbonne Universites, Museum national d’Histoire naturelle, CNRS, CP52, Paris, France; 3 Institute of Biodiversity Animal Health and Comparative Medicine, University of Glasgow, Glasgow, United Kingdom; National Institutes of Allergy and Infectious Diseases, NIH, UNITED STATES

## Abstract

**Background:**

The release of small non-coding RNAs (sRNAs) has been reported in parasitic nematodes, trematodes and cestodes of medical and veterinary importance. However, little is known regarding the diversity and composition of sRNAs released by different lifecycle stages and the portion of sRNAs that persist in host tissues during filarial infection. This information is relevant to understanding potential roles of sRNAs in parasite-to-host communication, as well as to inform on the location within the host and time point at which they can be detected.

**Methodology and principal findings:**

We have used small RNA (sRNA) sequencing analysis to identify sRNAs in replicate samples of the excretory-secretory (ES) products of developmental stages of the filarial nematode *Litomosoides sigmodontis in vitro* and compare this to the parasite-derived sRNA detected in host tissues. We show that all *L*. *sigmodontis* developmental stages release RNAs *in vitro*, including ribosomal RNA fragments, 5’-derived tRNA fragments (5’-tRFs) and, to a lesser extent, microRNAs (miRNAs). The gravid adult females (gAF) produce the largest diversity and abundance of miRNAs in the ES compared to the adult males or microfilariae. Analysis of sRNAs detected in serum and macrophages from infected animals reveals that parasite miRNAs are preferentially detected *in vivo*, compared to their low levels in the ES products, and identifies miR-92-3p and miR-71-5p as *L*. *sigmodontis* miRNAs that are stably detected in host cells *in vivo*.

**Conclusions:**

Our results suggest that gravid adult female worms secrete the largest diversity of extracellular sRNAs compared to adult males or microfilariae. We further show differences in the parasite sRNA biotype distribution detected *in vitro* versus *in vivo*. We identify macrophages as one reservoir for parasite sRNA during infection, and confirm the presence of parasite miRNAs and tRNAs in host serum during patent infection.

## Author summary

Lymphatic and visceral filariasis, as well as loiasis and onchocerciasis, are parasitic infections caused by filarial nematodes that can cause extensive and diverse clinical manifestations, including edemas of the lower limbs and visual impairment. These parasites successfully maintain a crosstalk with the immune system of their host and one potential mediator of this communication is extracellular small non-coding RNAs (sRNAs) released by the parasite. However, little is known of the mechanisms of sRNA export, how the exported sRNAs differ between lifecycle stages, and how the parasite microenvironment (e.g. *in vitro* vs. *in vivo*) contributes to the composition of sRNAs that can be detected. In this report, we show that all the developmental stages of the filarial parasite *Litomosoides sigmodontis* release sRNAs, which include tRNA fragments and miRNAs, *in vitro*. A subset of the miRNAs are differentially represented in the ES products between adult stages (males and gravid females) and larval stages (microfilariae) *in vitro*, however all of the miRNAs detected in serum or macrophages *in vivo* are present in the ES from all life stages. We show that the parasite-derived miRNAs are protected from degradation *in vitro* and are stable *in vivo*, as they are readily detectable in the serum of infected jirds. Several parasite miRNAs are also detected within macrophages purified from infected hosts, consistent with parasite RNAs having a yet unidentified functional role in host cells.

## Introduction

Filarial nematodes are the causative agents of human lymphatic filariasis (*Wuchereria bancrofti* and *Brugia malayi*), serous cavity filariasis (*Mansonella spp*.), Loiasis (*Loa loa*) and Onchocerciasis (*Onchocerca volvulus*). These debilitating neglected diseases of the tropics impose a heavy health and socioeconomical burden in developing countries in Africa, south east Asia, and Latin America [1,2]. Latest estimations suggest that more than 60 million people are infected and at least 40 million present clinical manifestations of either disease, with a further estimated 110 million infected by *Mansonella spp*., which is mostly asymptomatic but can reach 90% prevalence in the tropics [3–5]. Moreover, more than 900 million people are at risk of infection in endemic areas, making the control and elimination programs particularly challenging [1,2]. Importantly, the *in vitro* culture of parasitic nematodes, including filarial nematodes, has paved the way to understand many facets of the biology of these pathogens [6–9], thus facilitating the development of novel and more effective therapeutic approaches, as well as the mode of action of some of the most widely used treatments for filarial infections [4,10,11].

Over the last few years, miRNAs have emerged as another component of the Excretory/Secretory (ES) products of filarial nematodes, including dog heartworm *Dirofilaria immitis* [12] and the human parasite *B*. *malayi* [8]. Parasite-derived miRNAs have also been identified in the serum of mice infected with *Litomosoides sigmodontis* [13], dogs infected with *D*. *immitis* [14], baboons infected with *L*. *loa* [15], human patients infected with *O*. *volvulus* [14,16] and in the nodules of cattle infected with *O*. *ochengi* [15,16]. These studies suggest that filarial miRNAs are naturally released during infection, which has led to extensive interest in their biomarker capacity and in particular whether they could indicate the presence of viable adult females [17]. However, to date, very little has linked the observations of miRNAs *in vitro* with what is detected *in vivo* and, in general, little is known about the composition and stability of parasite miRNAs in either context. Furthermore, additional classes of extracellular RNA have been reported to exist in other parasitic animals, including tRNA fragments and siRNAs, which have not yet been investigated in filarial nematodes.

Here we characterize extracellular sRNAs in *L*. *sigmodontis*, a filarial nematode that naturally infects cotton rats and has been used extensively to decipher the molecular and immunological basis of filarial infections [18]. Following infection of the host, the infective L3 stage migrates from the site of infection in the skin to the pleural cavity [19], where it undergoes two consecutive molting steps into the adult stage which releases microfilariae into the bloodstream, where they become detectable around day 60 post-infection [20], making them available to a potential blood-feeding vector. Our previous report demonstrated the presence of *L*. *sigmodontis*-derived miRNAs in the blood circulation of BALB/c mice during the patent stage of the infection [13] when both adults and microfilariae are present in the host. Here we use *in vitro* approaches to differentiate the sRNAs released by the different lifecycle stages of the parasite. We further compare this to the full sRNA content detected in serum as well as macrophages from infected animals. Our results demonstrate that all life stages release sRNAs *in vitro*, and these are dominated by 5’-tRNA fragments, rRNAs, and to a lesser extent miRNAs. Statistical analysis of these datasets reveals specific profile differences between the miRNAs released from adult and larval stages and suggest that gravid adult female (gAF) worms release the largest diversity of miRNAs *in vitro*, when compared to either adult males or mf. At the same time, we identify marked differences between the parasite-secreted sRNA profile *in vitro* (mostly associated with 5’-tRNA halves and rRNA) and *in vivo*, where miRNAs are the most abundant class of parasite exRNA that can confidently be assigned to the parasite. Furthermore, we show that the parasite-derived miRNAs are not exclusively found in free circulation, but also within macrophages, consistent with the possibility that sRNAs mediate parasite-host communication [21–23].

## Materials and methods

### Ethical considerations

All animals were maintained under specific pathogen free (SPF) conditions at the University of Edinburgh Animal Facilities. Animal experiments were conducted under Project Licenses granted by the Home Office (United Kingdom), references 70/8548 and 70/8896, in accordance with local guidelines and approved by the Ethical Review Committee of the University of Edinburgh.

### *Litomosoides sigmodontis* lifecycle

*L*. *sigmodontis* was maintained by passage through 12-week-old male jirds (*Meriones unguiculatus*), and the arthropod intermediate host mite (*Ornithonyssus bacoti*), as previously described [24–26]. All infections for this study were conducted by intraperitoneal injections of the jirds, as previously reported [7]. For *in vitro* culture, all parasite developmental stages were cultured in RPMI-1640 medium culture supplemented with 100 U/mL penicillin, 100 μg/mL streptomycin, 1% D-(+)-glucose and 0.1 mg/mL gentamycin and 30 mM HEPES. For the adult stages, worms were cultured at a density of 1–2 worms/mL, whereas the larval stages were kept at a density of 250–500 larvae/mL, and the microfilariae at a density of 250,000–500,000 mf/mL.

### Generation of Excretory/Secretory (ES) products from larval and adult stages of *L*. *sigmodontis*

For the adult stages, the media was harvested every 24h for up to 72h. For larval stages, media was harvested after 72h in culture to avoid excessive handling of potentially fragile developmental stages. Harvested media was centrifuged at 1,500g for 20 min at room temperature and filtered through a 0.22 μm filter (Millex) to remove any debris and stored at -20°C until analyzed. For vesicle quantification, Nanoparticle Tracking Analysis (NTA) was carried out using the NanoSight LM14 instrument (Malvern Instruments, Malvern, UK). For purification of total RNA from adult lifecycle stages, an equivalent volume of ES products (~1–2 worms/ml) harvested at each time point (~30 mL per time point) were either pooled together or kept as discrete fractions of early (0-24h) and late (48-72h) ES products, concentrated to 1.5 mL using Vivaspin 6 centrifugal concentrators 5 kDa MWCO (Sartorius). Total RNA was purified using the miRNeasy mini kit (Qiagen) with the following modifications. Briefly, 1.5 volumes of concentrated ES product (pooled or as individual fractions) were incubated with 3.5 volumes of Trizol LS (ThermoFisher), vortexed for 30 seconds, and mixed with 1 volume of chloroform. The upper aqueous phase was then taken through the purifications recommended by the kit. The final elution was in 25 μl of nuclease-free distilled water. Small RNA content and quality was determined using 1 μl of total RNA on a Bioanalyzer small RNA kit (Agilent) prior to small RNA library preparation.

### *In vitro* viability assay (MTT) of gravid adult female worms

Adult worms were separated by sex as described elsewhere [7], and female worms were placed in 24-well plates at a density of 1 worm/well in 2 mL of supplemented worm culture medium, and incubated at 37°C and 5% CO_2_. At different time points, worms were incubated with 3-(4,5-Dimethylthiazol-2-yl)-2,5-Diphenyltetrazolium Bromide (MTT) (Life technologies) at final concentration of 0.5 mg/mL, as described previously [27]. The viability was assessed by the capacity of gAF worms to reduce tetrazolium (MTT) salt, rendering a blue color worm, and compared to the signal obtained for worms freshly harvested from infected jirds (“time 0h”).

### Dissection of gravid adult female worms

A total of >60 *L*. *sigmodontis* adult gravid female worms were recovered from infected jirds at day 90 post-infection. To recover the body wall, digestive, and reproductive tracts, worms were chilled at 4°C, and dissected using a stereomicroscope and fine tipped forceps as described previously [28]. The “body wall” is represented by the empty carcass after removing the digestive and the reproductive tissue. A single pooled sample per anatomical fraction (body wall, digestive, or reproductive) was collected in Trizol (Ambion) and then RNA extracted according to the manufacturers’ protocol. Total RNA was eluted in 50 μl of nuclease-free distilled water and quantified by Qubit 2.0 Fluorimeter (Invitrogen) and kept at -80°C.

### Collection of serum, pleural/peritoneal (PLEC/PEC) exudates, and macrophages from murine hosts

All the animals used in this study were culled by CO_2_ asphyxiation in accordance to the UK Home Office regulation. To isolate blood-circulating RNAs, animals were exsanguinated by cardiac puncture and blood was incubated for 1h at room temperature, centrifuged at 400xg for 10 min at room temperature and the supernatant was further cleared by centrifugation at 16,000xg at room temperature for 5 min prior to storage at -20°C. The detection and quantification of circulating microfilaria was carried out as indicated elsewhere [24,29]. For RNA extraction, 0.2 ml of naïve and infected sera was used as input for the miRNeasy mini Kit (Qiagen), following the manufacturer’s recommendations. Total RNA was eluted in 50 μl of nuclease-free distilled water. The recovery of the pleural and peritoneal (PEC/PLEC) washes was conducted as previously reported [24]. To harvest Pleural/Peritoneal (PEC/PLEC) macrophages from jirds, we followed the previously described protocol for harvesting monocytes/macrophages by adherence to plastic [30]. Briefly, we pelleted the total cell population at 400xg for 10 min at 4°C. The cell pellet was then resuspended in 1 mL of supplemented RPMI-1640 at a concentration of 1.5x10^6^ cells/ml, and 100 μL were seeded into 96-well round bottomed plates (~1.5x10^5^ cells/well). After an incubation of 1h at 37 ^o^C and 5% CO_2_, non-adherent cells and microfilariae were removed by extensive washing of the wells with 1X PBS (Sigma). Adhered cells were lysed using 1 mL of Trizol (Ambion) and total RNA was purified using the miRNeasy mini Kit (Qiagen), following the manufacturer’s recommendations. We note there are not bona fide antibodies against surface antigens for macrophages purified from jirds. We therefore phenotyped adherent BALB/c macrophages by flow cytometry using the following antibodies (all prepared in 1:200 dilution): anti-CD45.2 (hematopoietic lineage), anti-CD3 (T cells), anti-CD19 (B cells), Ly6G (neutrophils), SiglecF (eosinophils), Ly6C (pro-inflammatory monocytes), CD11c (dendritic cells), CD11b and F480 (macrophages). All the antibodies were purchased from BD Biosciences, except CD45.2 and Ly6C, which were sourced from BioLegend. With this method we recover 50–80% of CD11b^+^/F480^+^ cells. RNA samples were quantified by Qubit (Invitrogen) and stored at -80°C until small RNA library preparation and/or qRT-PCR analysis.

### Small RNA library preparation from ES products, serum and adherent macrophages

For the preparation of libraries containing 5’-polyphosphate RNAs, total RNA from ES products or gAF worms were treated with 20U 5’ Polyphosphatase (Epicenter) for 30 min followed by ethanol precipitation as recently described [31]. For the analysis of small RNA content by next generation sequencing, libraries were prepared using 2.5 μl of total RNA using the CleanTag small RNA Library Prep kit (TriLink), according to the manufacturer’s protocol, using a 1:12 dilution of both adapters. Following initial testing, the final PCR amplification was set at 22 cycles. PCR products of the expected molecular weight (140-160bp) were size selected and samples were sequenced on an Illumina HiSeq high output v4 50bp single-end by Edinburgh Genomics.

### Bioinformatic analysis

Unprocessed sequence data for each sample were analyzed by FASTQC to obtain an overview of the sequence data quality. Subsequently, the 3’ sRNA adapter was removed using cutadapt [32], searching for at least a 6-base match to the adapter sequence. For analysis of small RNAs, only sequences that contained the adapter, were >16 nt in length, and were present in ≥ 2 copies were retained for further analysis after generating a non-redundant sequence set for each sample. As the *M*. *unguiculatus* genome had not been sequenced at the time of this study, sequences were aligned to the mouse genome (mm10). Alignments were also performed using the *Wolbachia L*. *sigmondontis (Wlsi)* endosymbiont (http://nematodes.org/genomes/litomosoides_sigmodontis/), and the *L*. *sigmodontis* draft genome (v 2.1) [33] using bowtie [34], requiring perfect matches along the full length of the sequence. Combining the data from the alignments, we defined *L*. *sigmodontis*-specific sequences as those sequences that matched perfectly and unambiguously to the *L*. *sigmodontis* draft genome but not to *M*. *musculus* nor *wLsi*.

miRNAs were predicted using only *L*. *sigmodontis*-specific reads from all samples in **S2** and **S5 Tables**. We used miRDeep2 v2.0.0.8 [35] with default settings, utilizing mature and hairpin sequences from miRBase v22 [36] and additional nematode miRNA sequences from *Brugia pahangi* and *Haemonchus contortus* [37]. miRDeep2 predicted a total of 376 miRNAs but we discarded 240 low confidence predictions flagged as rRNA/tRNA or with a miRDeep2 score <1, or that were present at <100 reads. miRNA names were assigned using known miRBase names in a hierarchical fashion, with highest priority assigned to precursors that matched perfectly to known nematode clade III miRNA precursors. If these did not exist, we named the miRNAs according to exact seed matches in mature sequences (positions 2–7) to known nematode mature miRNAs. miRNA predictions without a mature seed match to a known nematode mature miRNA were labelled novel and these were examined manually and discarded if their structure and read mapping profile did not fit expected miRNA criteria [38].

The *L*. *sigmodontis* draft genome was already annotated with protein-coding genes [39] but we added additional annotations to the genome: tRNA genes using tRNAscan-SE v1.3.1 [40]; rRNA and other Rfam genes using Infernal cmsearch v1.1.2 [41] with Rfam v13.0 covariance models [42]; repeat regions using RepeatModeler v1.0.11 (http://www.repeatmasker.org) on the draft genome followed by RepeatMasker v4.0.7 (http://www.repeatmasker.org) using RepBase v20170127 nematoda sequences [43]. Small RNA reads from each sample were mapped to the *L*. *sigmodontis* genome and annotated as miRNA, exonic, rRNA, tRNA, otherRfam, or repeats (in that order).

Pairwise comparisons between lifecycle stages from which we have more than two biological replicates were carried out to detect differentially expressed miRNAs using the Bioconductor DESeq2 package [44], with two of DESeq2’s heuristics disabled: independentFiltering = FALSE, cooksCutoff = FALSE since these heuristics are designed for dealing with large numbers of genes with large variations in counts. To ensure the statistical robustness of these analyses, we focused only on the ES products from developmental stages from which we had a considerable number of replicates; that is, adult males (AM), gravid adult females (gAF), and microfilariae (mf). As a cutoff, we only analyzed samples that had >80,000 *L*. *sigmodontis*-specific reads. Significance was assessed as having an experiment-wide false discovery rate (FDR) <0.05 (calculated using the Benjamini Hochberg method). For serum and macrophage samples sequenced from murine hosts, we mapped all reads from these samples to a combined set of all murine miRNA sequences from miRBase v22 and 120 *L*. *sigmodontis* miRNAs.

To determine which sRNA reads originated from tRNA 5’ fragments in host tissue samples, we mapped reads > = 30 bp to the first 32 bp of all tRNA sequences from both the host [45] and the parasite using bowtie with no mismatches. We confirmed that no host (murine) and parasite (*L*. *sigmodontis*) tRNA genes had the first 40 bp in common. If two tRNA genes were identical over the first 40 bp, we selected the first by alphabetical order to prevent double counting any tRNA reads.

### Detection of miRNAs by qRT-PCR

For reverse transcription of RNA from ES, a fixed volume of 2.5 μL of total RNA, (corresponding to 150 μL of concentrated ES) was used as input and 1 μL of 0.1 pM of the synthetic RT1 (**S1 Table**) was spiked into the reverse transcription reaction mix for normalization as indicated in the text. Normalization with the exogenous synthetic sRNA accounts for variation in qRT-PCR efficiency across samples but does not provide a control for RNA recovery (it is a challenge in general to identify suitable normalizers in extracellular samples [46] and we have not addressed this issue here). For analysis on gravid adult female worm tissues, 50 ng of total RNA was used. Reverse transcription reactions were performed using the miScript RT II System (Qiagen) according to the manufacturer’s protocol. Quantitative PCR was carried out with the QuantiTec SYBR Green PCR kit (Qiagen), which includes a universal primer, according to the manufacturer’s protocol. Primers for *L*. *sigmodontis* miRNAs were purchased from Invitrogen or IDT (**S1 Table**). Preliminary data, using total RNA from gravid adult female worms, confirmed that these primers amplified their targets with an average of 80–100% efficiency (**S1 Table**). For each sample, two technical replicates were included, as well as a nuclease-free water sample (“no template sample”) control to determine background signal. The relative expression was calculated using the 2^-ΔCt^ formula, where ΔCt represents the normalized Ct value of the target RNA relative to the signal of spiked RT1 [47] or to 18S rRNA (for tissues from gravid adult females). Statistical analysis was conducted using the Mann-Whitney test and *p* values <0.05 were considered statistically significant.

### RNase sensitivity analyses

To test the stability of released sRNAs *in vitro*, a total of 40 μL of concentrated ES from gAF worms was mixed with RNace-IT Ribonuclease cocktail (Agilent) in the absence or presence of 5 μg/μL Proteinase K (Epicentre) and 0.05% Triton X-100 (Merck). Incubations were conducted at 37°C for 30 min. RNA was then extracted with 5X volumes of Qiazol and purified by miRNeasy plasma/serum kit following the manufacturer’s recommendations and eluted in 16 μL of RNase-free water. 10 μL of total RNA was used for analysis by qRT-PCR using miScript II RT System (Qiagen) as indicated above.

### Statistical analysis

Statistical analyses were performed using Prism 7 (GraphPad Software Inc, USA). Initially, qPCR data were tested for normality using statistical packages built in Prism 7 (D’Agostino & Pearson normality test or Shapiro-Wilk normality test). If sample distributions conformed to normality groups, they were then compared using a Student’s unpaired *t* test. Otherwise, non-parametric Kolmogorov-Smirnov or Mann-Whitney tests were used to compare cumulative distributions or ranks, respectively. The information regarding the specific statistical analysis conducted in each experiment is provided in each figure legend.

## Results

### Profile of sRNAs from excretory-secretory (ES) products is distinct from that of adult *L*. *sigmodontis* worms and is dominated by tRNAs and rRNAs

**Fig 1:**
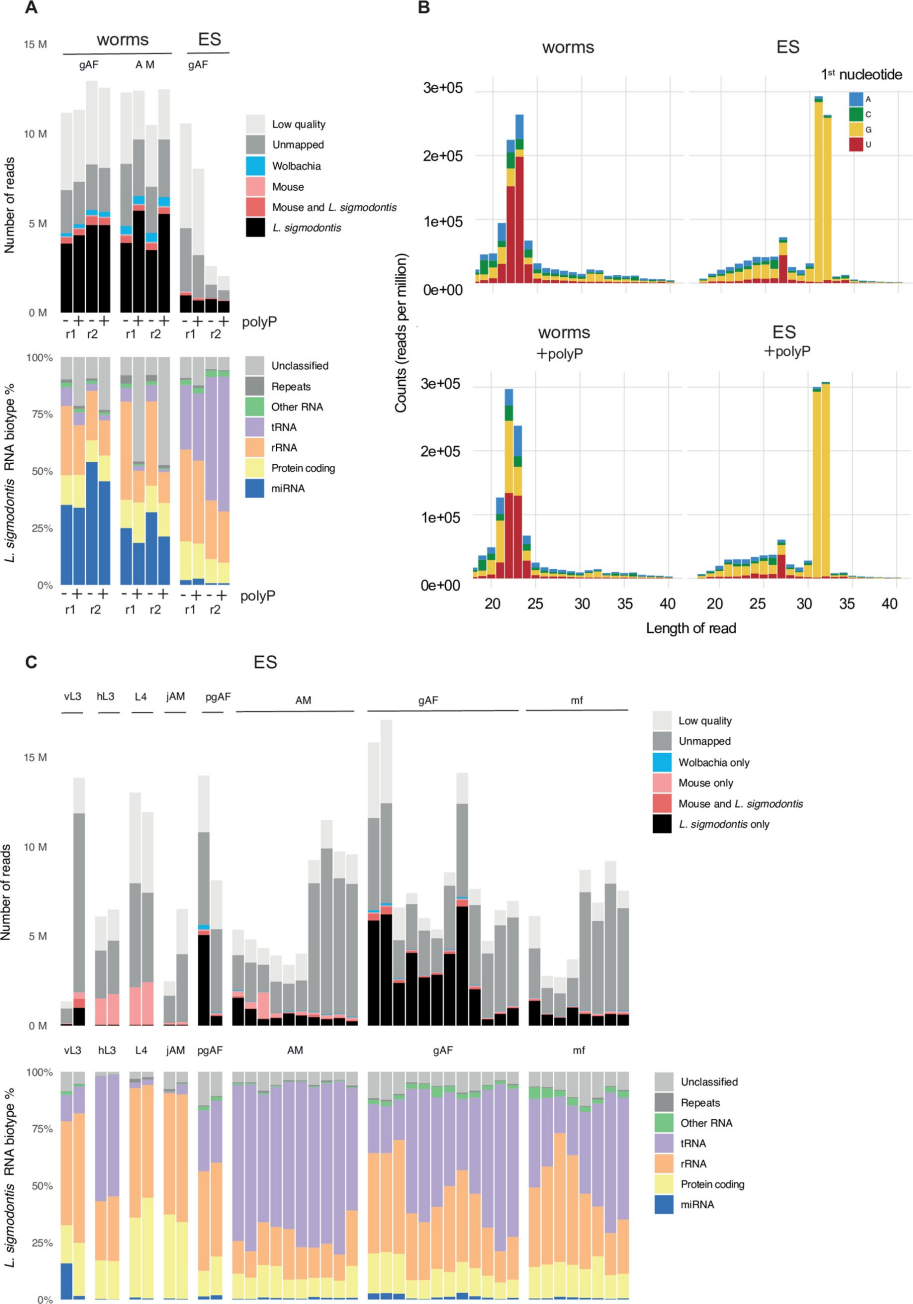
Composition of ES in *L*. *sigmodontis* adult worms compared to ES products of all lifecycle stages. **(A)** Composition of the sRNA sequencing libraries made in the absence (-) or presence (+) of polyphosphatase (polyP) from male (AM) and gravid adult female (gAF) worms in comparison to the ES of gAFs. Top panel: legend qualifies reads that are too short or reads with no adapters (light gray), reads that do not map to any of the genomes used as references in this study (dark gray), as well as reads mapping unambiguously to either the *M*. *musculus* genome (light pink), the *L*. *sigmodontis Wolbachia* (*wLsig)* endosymbiont genome (blue), or the *L*. *sigmodontis* genome (black); we also note the proportion of reads that map to both *M*. *musculus* and *L*. *sigmodontis* (dark pink). Lower panel: RNA biotype distribution of the reads that map unambiguously to *L*. *sigmodontis*. Two biological replicates (denoted as “r1” and “r2”) are shown for comparison. **(B)** First nucleotide and length of sRNAs made in the absence (top) and presence (bottom) of polyphosphatase for adult female worms (**left**) and ES products from adult females (right), each representative of two independent samples. **(C)** Top panel: Composition of the sRNAs in sequencing libraries (made in the absence of polyphosphatase) from the ES of the larval and adult lifecycle stages of *L*. *sigmodontis* isolated from either infected mites or jirds, as detailed in materials and methods. The legend defines read type as in (A). Lower panel: RNA biotype distribution of the reads that map unambiguously to *L*. *sigmodontis*. vL3s = vector-derived L3 stage (*n* = 2); hL3s = host-derived L3 stage harvested at day 3 post-infection (*n* = 2); L4 = 4^th^ stage larvae harvested at day 20 post-infection (*n* = 2); jAM = juvenile males and pgAF = pre-gravid adult females, harvested at day 32 post-infection (*n* = 2 for each sex); AM = Adult males (*n* = 10) and gAF = gravid adult females (*n* = 12), harvested at day 90 post-infection; mf = microfilariae (*n* = 8), harvested *in vitro* from gAF.

To capture the full range of sRNAs in the ES products of *L*. *sigmodontis* and include in the analysis sRNAs harboring 5’ triphosphates, RNA was prepared in the absence and presence of polyphosphatase treatment prior to library preparation. To identify sequences likely to be derived from the host we used the murine genome (as no reference genome was available for *M*. *unguiculatus* at the time this work was being performed). High quality reads were classified as mapping perfectly to either *L*. *sigmodontis*, the *L*. *sigmodontis Wolbachia* (*wLsig)* endosymbiont, or the *M*. *musculus* reference genomes (**Fig 1A**). The proportions assigned to each genome are variable across samples and likely to reflect variation in the amount of *bona fide L*. *sigmondontis* RNA in each sample (we infer that reads mapping to the host could be contamination, although these are not examined here). In general, across all libraries we detect a higher % of reads derived from *L*. *sigmodontis* in the adult worms versus the ES products (**Fig 1A** and **S2 Table**). Of the reads that map to *L*. *sigmodontis*, there is a larger proportion of miRNAs in the adult libraries compared to ES products (**Fig 1A** and **S2 Table**). Consistent with this, plotting the length and first nucleotide preference of the sRNAs in adults reveals an enrichment of sequences 22 nt in length with a 1^st^ nucleotide of uracil, characteristic of mature miRNAs. This enrichment is not obvious in the ES products (**Fig 1B**). Treatment of the RNA with polyphosphatase prior to library preparation showed an increase in 22 nt RNAs with 1^st^ nucleotide of guanine (**Fig 1B**). This pattern is consistent with secondary siRNAs produced from RNA-dependent RNA polymerases (RdRPs), as previously reported for *Caenorhabditis elegan*s, *B*. *malayi*, *Heligmosomoides bakeri*, and *Strongyloididae* species [31,48–50]. In contrast, the small RNA profile of the ES products show a distinct pattern compared to the adult worms, with only minor qualitative differences between the monophosphate and polyphosphate libraries and a peak of longer reads (~31–32 nt) in the size range of tRNA fragments (**Fig 1B**). These results suggest secondary siRNAs are not a dominant feature in *L*. *sigmodontis* ES products, in contrast to what is found in the extracellular vesicles (EVs) of Clade V nematodes [31].

To examine and compare the sRNA content released from all life stages, datasets were generated from the *in vitro* ES products from larval (vector-derived L3s - vL3s, host-derived L3s - hL3s, L4s, and mf) and adult stages (juvenile males–jAM, pre-gravid females–pgAF, adult males–AM, and gravid adult females–gAF). In the absence of other metrics, we extracted RNA from equivalent volumes of ES from each stage, as described in materials and methods. The small RNA content of the ES products, defined as the population of total RNA <150 nt in length, showed two main populations, 20–30 nt in length and 40–60 nt in length (**S1 Fig**), as described previously in other filarial models [16]. Clear differences in the signal intensities and concentrations reported by the instrument were observed, likely reflecting different total RNA levels in the ES products of each stage. Reads mapping to both *M*. *musculus* and *L*. *sigmodontis* are qualified and displayed in **Fig 1C** (dark pink) and documented in **S2 Table** but were not used for subsequent analysis, where we focused exclusively on reads that could be confidently assigned to the parasite. This analysis identified varying percentages of *L*. *sigmodontis*-specific reads in ES products: 12.2% from vL3s, 1.5% in hL3s, 0.94% in L4s, 2.46% in jAM, 35.5% in pgAF, 14.1% in AM, 43.5% in gAF, and 13.4% in mf (**Fig 1C & S2 Table**). We detected reads mapping to the *Wolbachia* endosymbiont in the adult libraries in particular, and to a lesser extent in all datasets (**Fig 1 & S2 Table**), however it is noted that these may also map to other bacteria. We therefore cannot confirm at this stage whether *Wolbachia*-derived RNAs are present in ES products, since bacterial reads can contaminate small RNA libraries [51,52]. We note that >25% of the total high-quality reads in hL3s and L4s mapped perfectly and unambiguously to the *M*. *musculus* genome, which contrasts with the <8% total *M*. *musculus*-specific reads detected in ES products of the remaining lifecycle stages (**Fig 1C & S2 Table)**. A small proportion of the *L*. *sigmodontis*-specific reads could not be classified as the canonical families reported in Rfam, and further investigation revealed these mapped to either exon sequences, repetitive elements, or did not match any known RNA biotype (“unclassified”) (**Fig 1C & S2 Table**). Taken together, these results provide a global analysis of the content of a small RNAs in ES products and clearly demonstrate that both larval and adult worms release sRNAs *in vitro* that are dominated by rRNAs and tRNAs, and miRNAs to a lesser extent.

### 5’-derived tRNA fragments are abundantly detected in the ES products of *L*. *sigmodontis in vitro*

**Fig 2:**
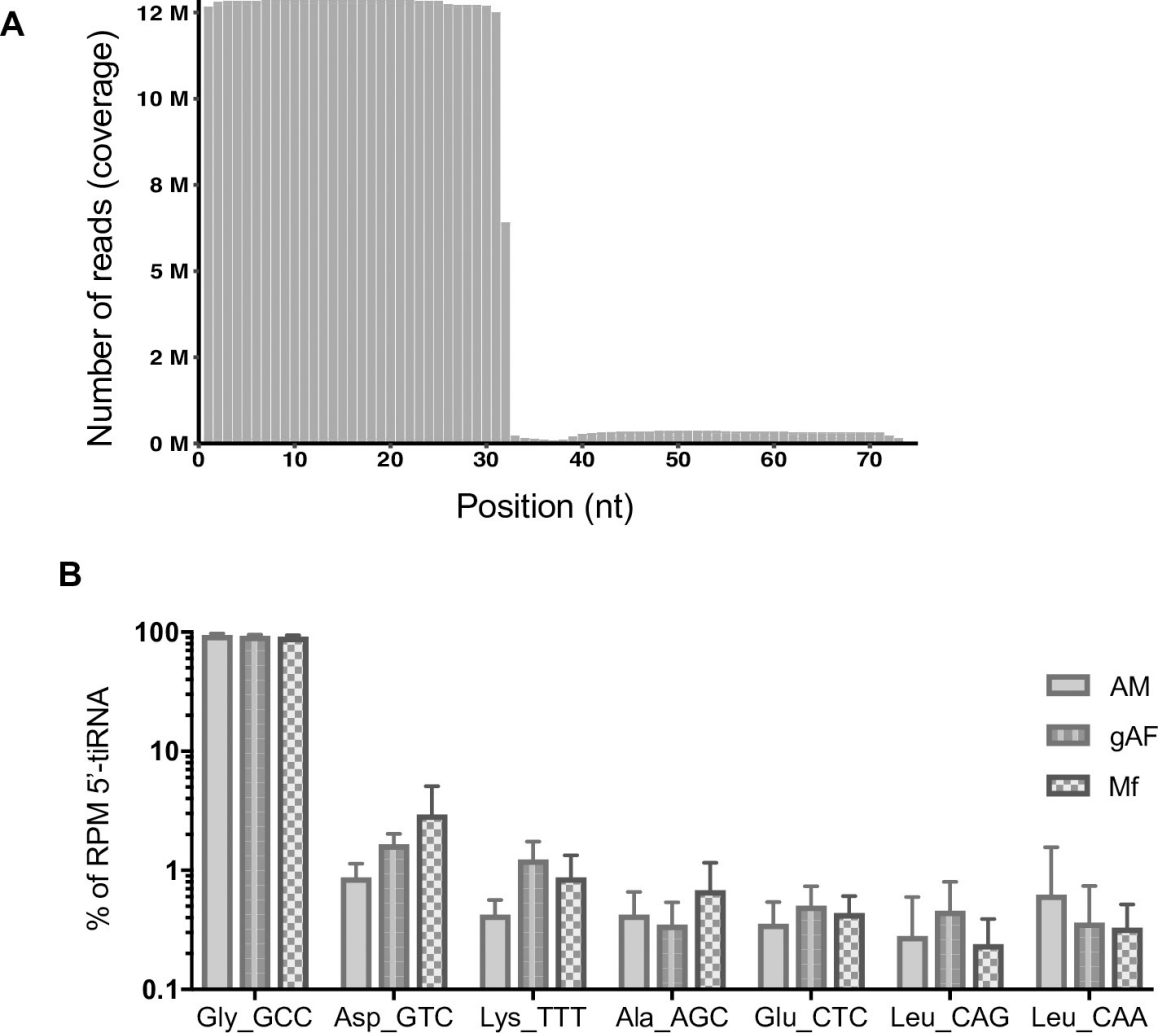
5’-derived tRNA fragments dominate the ES products of *L*. *sigmodontis* adults and microfilariae. **A)** Length distribution of reads mapping to putative *L*. *sigmodontis* tRNAs in ES products from gravid adult females (gAF) worms. **B)** The top seven most abundant tRNA fragments in ES products from Adult males (AM), gravid adult females (gAF), and microfilariae (mf), representing >95% of the total *L*. *sigmodontis*-specific 5’-tRNA counts.

Recently reports across diverse organisms demonstrate tRNA fragments are a ubiquitous component of extracellular environments. To accurately characterize the tRNA products in ES we defined the tRNA content of the *L*. *sigmodontis* genome draft using tRNAScan-SE [40] which identified 20 canonical tRNA genes from 65 different loci (**S3 Table**). Mapping of the tRNA sequences from ES products shows a clear enrichment for starting at the first nt of the mature tRNA and spanning 30–35 nts (**Fig 2A**), consistent with what has been defined in other organisms as a tRNA fragment (tRF-5) or tRNA half (**Fig 2A and S2A Fig**) [53,54]. The top most abundant fragments (>10,000 reads per million tRNA read counts) detected in the ES products of AM, gAF, and mf are summarized in **Table 1** and **S4 Table**. Most of the tRNA reads across the ES samples map to tRF-Gly-GCC (**Fig 2B and Table 1**) and accounted for ~60% of the total tRNA reads identified in our study (**S4 Table)**, which could relate to recent findings that fragments of tRNA Gly are highly stable due to dimerization [55]. Several other tRNA fragments identified here ranked differently in the ES products of different developmental stages of *L*. *sigmodontis*; for example, tRNA-Ala-AGC and tRNA-Leu-CAA ranked higher in ES products from AM, whereas tRNA-Lys-TTT ranked higher in ES products from both gAF and mf (**Table 1**). These results suggest potential stage- and/or sex-dependent differences in the secreted tRNA fragments detected *in vitro*, which requires further investigation.

**Table 1:** Ranked list of the top 15 most abundant 5’-tRF detected in the ES products of *Litomosoides sigmodontis*.

tRNA	Anticodon	Genomic locus	RNA Sequence (5’– 3’)	Rank AM ES products (*n* = 10)^1^	Rank gAF ES products (*n* = 12)^1^	Rank mf ES products (*n* = 8)^1^
**tRNA-Gly**	GCC	nLs.2.1.scaf00610	acgcgggcggcccggguucgauucccggccgaugc	1	1	1
**tRNA-Asp**	GTC	nLs.2.1.scaf00398	acgugcgagacccggguucgauucccggccggggag	2	2	2
**tRNA-Ala**	AGC	nLs.2.1.scaf00005	augggagagggcugggguucgauuccccauauc	3	5	4
**tRNA-Leu**	CAA	nLs.2.1.scaf00177 nLs.2.1.scaf00175	gaggagugugacaugggcguucugguaucguaaucg	4	10	9
**tRNA-Leu**	AAG	nLs.2.1.scaf00506 nLs.2.1.scaf00702	cuaaggcgcugguuuaaggcaccagucucuucgggggcgugg	5	12	13
**tRNA-Lys**	TTT	nLs.2.1.scaf00175 nLs.2.1.scaf00072	gccuucuuagcucagucgguagagcaucagac	6	3	3
**tRNA-Glu**	CTC	nLs.2.1.scaf00777	cccgcaaggcccggguucaauucccggcaacggaa	7	4	5
**tRNA-Ala**	TGC	nLs.2.1.scaf00175 nLs.2.1.scaf00398	cgcuuugcaugcgagagggcggggguucgauccc	8	13	10
**tRNA-Leu**	CAG	nLs.2.1.scaf01110 nLs.2.1.scaf00447	cgguaggcgcagguucgaauccugcggcggac	9	6	11
**tRNA-Lys**	CTT	nLs.2.1.scaf00729 nLs.2.1.scaf00389 nLs.2.1.scaf00801	gguuagcucagucgguagagcaccagacucuuaaucug	10	9	15
**tRNA-Trp**	CCA	nLs.2.1.scaf00704	gaagguugcguguucgaaucgcgucgggguca	11	14	12
**tRNA-Val**	AAC	nLs.2.1.scaf00486 nLs.2.1.scaf00175	ggucucgugguguagcgguuaucacaucuguc	12	7	6
**tRNA-Gln**	CTG	nLs.2.1.scaf00353 nLs.2.1.scaf00610	cucaggacucugaauccugcgacgagaguucaa	13	11	7
**tRNA-Glu**	TTC	nLs.2.1.scaf00695	gcuaggauucguggcuuucacccacgcggcccgggu	14	8	8
**tRNA-Ala**	CGC	nLs.2.1.scaf00004	ggagaggucugggguucgauuccccaugccucca	15	15	14

The 5’-derived tRNA fragments reported here are those with a relative abundance of >1,000 RPM.

^1^ The median of the sequencing reads of the top most abundant 5’-derived tRNA fragments in the ES products of adult males (AM), gravid adult females (gAF), and microfilariae (mf) was used to determine the ranked position.

### Differentially detected miRNAs in the ES products of *L*. *sigmodontis*

**Fig 3:**
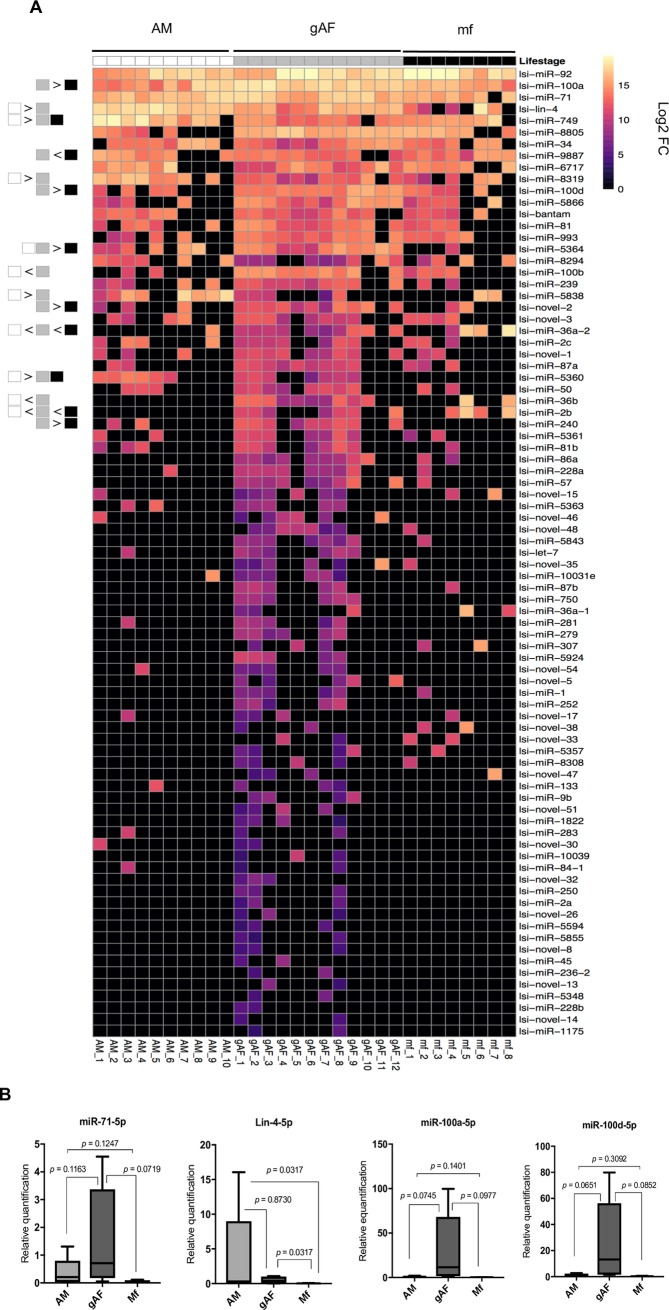
miRNA identification in the ES products of *L*. *sigmodontis*. **A)** *L*. *sigmodontis* miRNAs identified in ES products in > 2 samples, normalized as reads per million (RPM) to the total miRNA reads in each sample, represented in log2 scale. The heat map is organized per sample and group type as AM (white, left), gAF (light gray, middle) and mf (black, right). The boxes at the far-left section of the heat map represent the pairwise differential expression determined by DESeq2 analysis, with boxes being color-coded as indicated, and the symbols “>” or “<” shown if there is a significant difference in enrichment levels of that miRNA in ES from one stage versus the other. The symbol “>” indicates that the lifecycle stage(s) on the left have higher expression than the lifecycle stage(s) on the right, and vice-versa for “<” (adjusted p value <0.05). **B)** Relative expression of miRNAs in ES products of the different lifecycle stages based on qRT-PCR (using RNA extracted from equal volume of ES), normalized to RT1; Y axis is shown as log scale. miRNAs shown are: predicted to be commonly detected in all ES products (miR-71-5p), enriched in the ES products of AM (Lin-4), or enriched in the ES products from gAFs (miR-100a-5p and miR-100d-5p), see **Table 3**. *P* values were calculated using either Student’s unpaired t test or Kolmogorov-Smirnov test, for parametric and nonparametric data distributions, respectively. Statistical significance was considered when *p* value <0.05, and a trend is mentioned in text when *p* values < 0.1. For qRT-PCR analyses, the following number of replicates of ES products were used: AM (adult males), *n* = 5; gAF (gravid adult females), *n* = 5; mf (microfilariae), *n* = 4.

To identify all miRNAs in the ES products we used miRDeep2 [56] with *L*. *sigmodontis*-specific reads as input, including those derived from the adult worms. **S5 Table** shows the counts across all lifecycle stages for the 120 *L*. *sigmodontis* miRNAs identified (see Methods for details). Of these, 23 miRNAs were present only in the adult worms, with no reads mapping to these miRNAs in the ES samples. The 20 most abundant miRNAs detected in ES products across developmental stages account for ~95% of the total miRNA reads quantified by miRDeep2 (**Table 2)**. Fig 3A shows a heat map of the ES miRNA reads in the lifecycle stages for which there were sufficient *L*. *sigmodontis* reads: adult male (AM), gravid adult female (gAF) and microfilaria (mf). Only 83 miRNAs with counts in at least two of the AM, gAF and mf samples are shown and the differentially expressed miRNAs are noted (**Fig 3 and Table 3**). The ES products from gAF worms contain a greater diversity of miRNAs at this level of detection (**Fig 3**). The most abundant miRNA family detected was the miR-10 family, with four members (miR-100a-5p, miR-100b-5p, miR-100d-5p and miR-993-3p) representing ~17% of the total miRNAs identified by miRDeep2 (**Table 2**). These miRNAs share the same seed sequence, defined as nucleotides in position 2–7 from the 5’ end, and thus are assigned as members of the same family (**S3A Fig**). Beyond the conserved families, 28 novel miRNAs (out of 42 identified) made the criteria for inclusion in DESeq2 analysis (**S5 Table**) and these accounted for ~1.4% of the total miRNA counts. Since these do not have homology to known nematode miRNAs, their origins and evolutionary history are less well understood. However, they are predicted to represent *bona fide* dicer products from their stem-loop structures and 3’ overhangs, as shown in the top three most abundant predicted miRNAs (**S3B Fig**).

**Table 2:** Top 20 most abundant miRNAs detected in the ES products of *Litomosoides sigmodontis*.

miRNA	Mature RNA sequence (5’– 3’)	RPM^1^	Reads in AM ES products (*n* = 10)^2^	Reads in gAF ES products (*n* = 12)^2^	Reads in mf ES products (*n* = 8)^2^
**lsi-miR-100a-5p**	aacccguaguuucgaacaugugu	236,187	81	5,562	188
**lsi-miR-92-3p**	uauugcacucgucccggccuga	183,225	236	5639	1292
**lsi-miR-71-5p**	ugaaagacauggguagugagacg	179,955	285	2,578	205
**lsi-lin-4-5p**	ucccugagaccucugcugcga	61,615	443	1,465	8
**lsi-miR-8805-5p**	ggaggaaucagcgugcugu	60,186	5	2,452	208
**lsi-miR-749-5p**	gccuggaugaaucucggug	32,853	249	198	141
**lsi-miR-5364-3p**	cgagguauuguuuauuggcuga	27,242	7	329	0
**lsi-miR-100b-5p**	aacccguagauccgaacuugugu	23,526	0	749	6
**lsi-miR-5866-3p**	uuaccauguugaucgaucucc	21,567	0	206	13
**lsi-miR-81-3p**	ugagaucauugugaaagcuauu	20,342	7	177	9
**lsi-miR-993-3p**	uaagcucgucucuacaggcagg	14,878	0	213	29
**lsi-miR-100d-5p**	uacccguagcuccgaauaugugu	14,550	0	585	25
**lsi-miR-9887-5p**	cagggcugcacgcgcgc	12,566	52	277	62
**lsi-miR-34-5p**	uggcagugugguuagcugguugu	11,760	34	252	53
**lsi-bantam-3p**	ugagaucacguuacauccgccu	11,656	21	113	21
**lsi-miR-8319-3p**	gaauuagcucgugcgguacggc	8,553	111	118	10
**lsi-miR-36b-5p**	cgguacaacguuucacgguagagc	7,669	0	17	0
**lsi-novel-2-5p**	acgaugacaguaauaggauuauu	7,114	0	96	0
**lsi-miR-6717-5p**	gggcgaugaugguaugaagggua	6,784	74	244	101
**lsi-miR-5838-3p**	ugaguauuuucgguuucgcauc	6,234	80	0	0

^1^ RPM = Reads per million *L*. *sigmodontis* miRNA reads in all the sequencing libraries combined.

^2^ The median of the sequencing read counts is reported for AM, gAF and mf

**Table 3:** Differentially expressed miRNAs in the ES products of larval and adult stages of *L*. *sigmodontis*.

miRNA	Mature miRNA sequence	Compared to ES products from	Log2 FC	Adj *p* value (<0.05)	Previously reported *in vitro* and/or *in vivo* in other filarial infections?	References
**Enriched in ES products from gAF**						
lsi-miR-5364-3p	cgagguauuguuuauuggcuga	mf	6.01	7.8E-06	*O*. *volvulus*, *O*. *ochengi*, *D*. *immitis*, *B*. *malayi*	[8,12,14,16]
lsi-miR-240-3p	uacuggccuuucaaacucuaga	mf	5.28	5.1E-03	Not reported	
lsi-novel-2-5p	acgaugacaguaauaggauuauu	mf	3.30	3.9E-02	Not reported	
lsi-miR-100d-5p	uacccguagcuccgaauaugugu	mf	2.48	1.9E-02	*L*. *sigmodontis*, *O*. *volvulus*, *O*. *ochengi*, *D*. *immitis*, *L*. *loa*, *B*. *malayi*	[8,12–16]
lsi-miR-100a-5p	aacccguaguuucgaacaugugu	mf	1.41	3.7E-02	*L*. *sigmodontis*, *O*. *volvulus*, *O*. *ochengi*, *D*. *immitis*, *L*. *loa*, *B*. *malayi*	[8,12–16]
lsi-miR-36b-5p	cgguacaacguuucacgguagagc	AM	5.38	5.5E-04	Not reported	
lsi-miR-2b-5p	agcuguauuggcugugauaug	AM	4.63	3.3E-02	*B*. *malayi*	[8]
lsi-miR-100b-5p	aacccguagauccgaacuugugu	AM	4.44	1.7E-03	*B*. *malayi*	[8]
lsi-miR-36a-5p	cuggaugugcaucgugguugaug	AM	4.35	5.5E-03	Not reported	
**Enriched in ES products from AM**						
lsi-miR-5360-5p	acgaaucgucgaaucggauuuuu	mf	8.65	1.1E-03	*O*. *ochengi*, *D*. *immitis*, *B*. *malayi*	[8,12,14,16]
lsi-miR-5364-3p	cgagguauuguuuauuggcuga	mf	6.91	1.1E-02	*O*. *volvulus*, *O*. *ochengi*, *D*. *immitis*, *B*. *malayi*	[8,12,14,16]
lsi-miR-749-5p	gccuggaugaaucucggug	mf	3.70	4.6E-02	Not reported	
lsi-miR-5838-3p	ugaguauuuucgguuucgcauc	gAF	6.06	1.2E-04	*O*. *ochengi*	[16]
lsi-miR-749-5p	gccuggaugaaucucggug	gAF	4.82	1.2E-05	Not reported	
lsi-miR-8319-3p	gaauuagcucgugcgguacggc	gAF	4.10	3.9E-04	Not reported	
lsi-miR-5360-5p	acgaaucgucgaaucggauuuuu	gAF	3.80	2.5E-02	*O*. *ochengi*, *D*. *immitis*, *B*. *malayi*	[8,12,14,16]
lsi-lin-4-5p	ucccugagaccucugcugcga	gAF	2.55	3.9E-04	*L*. *sigmodontis*, *O*. *volvulus*, *O*. *ochengi*, *D*. *immitis*, *L*. *loa*, *B*. *malayi*	[8,12–16]
**Enriched in ES products from mf**						
lsi-miR-36a-5p	cuggaugugcaucgugguugaug	gAF	6.69	8.4E-04	Not reported	
lsi-miR-2b-5p	agcuguauuggcugugauaug	gAF	5.28	3.7E-02	*B*. *malayi*	[8]
lsi-miR-9887-5p	cagggcugcacgcgcgc	gAF	2.68	3.7E-02	Not reported	
lsi-miR-2b-5p	agcuguauuggcugugauaug	AM	24.71	3.8E-20	*B*. *malayi*	[8]

Although most miRNAs are detected in the ES from multiple lifecycle stages, 15 of the *L*. *sigmodontis* miRNAs showed a significant enrichment for one stage versus another based on pairwise comparisons (using an adjusted *p* value cut-off <0.05, **Table 3)**. For example, miR-5838 and lin-4 are enriched in ES of males compared to ES of gAF and Mf and miR-5364 is enriched in ES of adults (male or female) compared to ES of Mf (**Table 3**). Interestingly, lin-4 was also abundant in the ES products of *D*. *immitis* AM worms [12]. Similarly, 9 miRNAs were enriched in the ES products of gAF (e.g. miR-100a-5p, miR-100b-5p, among others) compared to AM and mf (**Table 3**). Some of these miRNAs were further examined by qRT-PCR, which revealed a consistent detection of miR-71-5p in all the ES products tested, an enrichment of Lin-4 in the ES products of AM, and a greater signal of miR-100a and miR-100d in the ES products of gAF when compared to AM and mf (**Fig 3B**). However, this failed to reach statistical significance and we note that there could still be a degree of cross-reactivity with contaminating host sequences (**Fig 3B**). We also note that the comparison of sRNA levels by qRT-PCR is not normalized to the total RNA present in each sample and given the larger quantities of RNA in the ES of gAF (**S1 Fig**), it is expected that the signals may be higher in these samples. Taken together, our sequencing data suggest that a subset of the miRNAs detected in the ES products of *L*. *sigmodontis* are enriched in a sex- and developmental stage-specific manner but most of them are ubiquitous to the ES products of all lifecycle stages.

### The sRNAs in ES from *L*. *sigmodontis* gravid adult female worms are stable 24–72 hours following release *in vitro* and protected from RNase degradation

**Fig 4:**
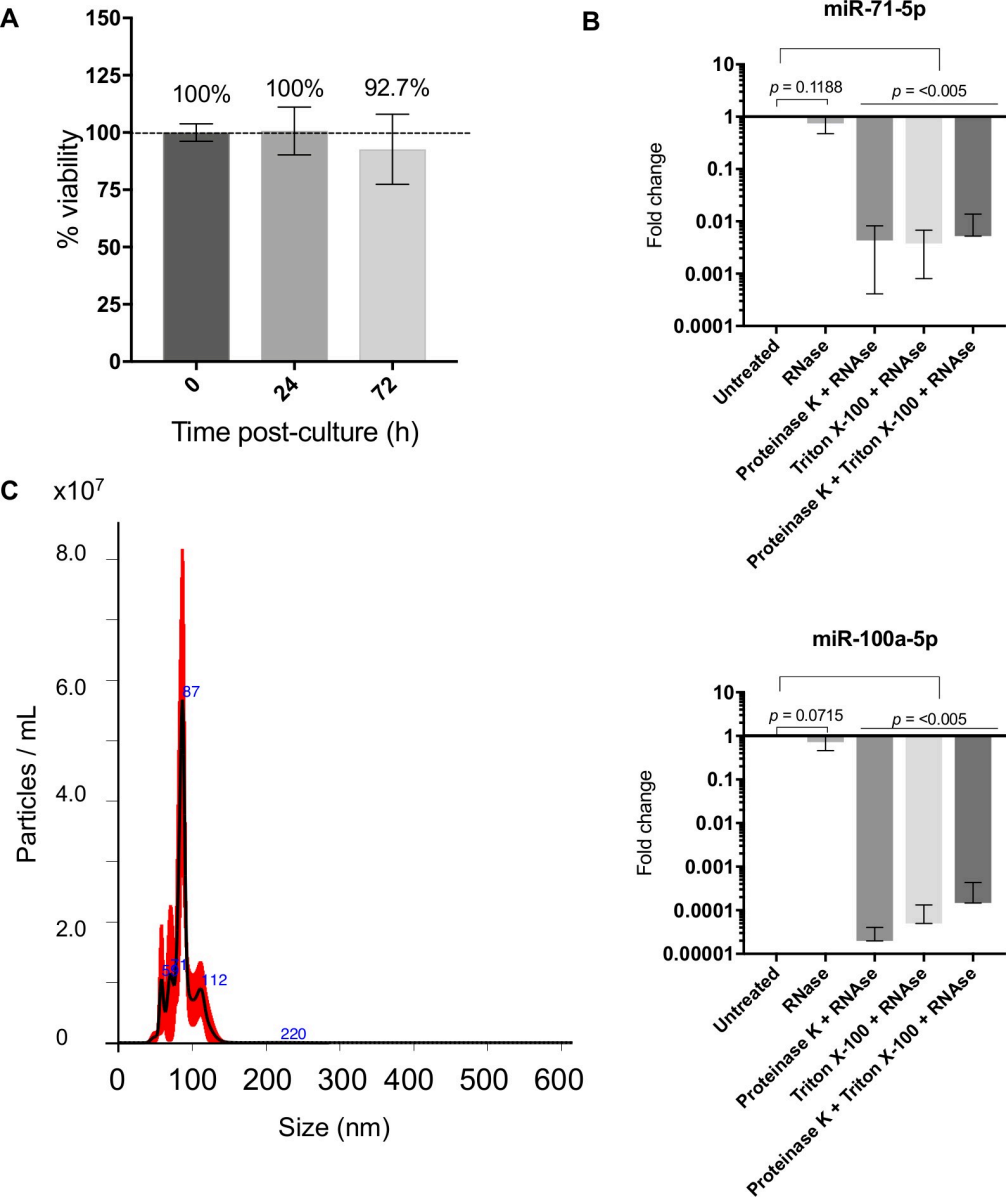
Viable gravid adult females secrete EVs and small RNAs that are protected from degradation. **A)** Viability of gAF worms over time *in vitro* (*n* = 2 independent experiments) reported as average ± standard deviation. **B)** RNase sensitivity of RNAs of miR-71-5p and miR-100a-5p (detected in the ES from gAFs) in the absence and presence of proteinase K (5 μg/uL) and Triton X (0.05%) for 30 min at 37°C. Results are reported as average ± standard deviation (n = 4). *P* values were calculated using either Student’s unpaired t test or Kolmogorov-Smirnov test using the “untreated” experimental group as a reference; *p*<0.05 was considered statistically significant. **C)** Size measurement of the extracellular vesicles detected in the ES from gAFs using NanoSight particle tracking (NTA) system.

A previous study has shown that extracellular vesicles (EVs) released by *B*. *malayi* L3s decreased in a time-dependent manner, perhaps owing to reduced worm viability over time [8]. Other reports have shown that *B*. *malayi* adult female worms respond transcriptionally to the *in vitro* culture conditions [57], although it unknown whether these transcriptional changes would be reflected in the small RNAs released. To better understand if the *L*. *sigmodontis* adult female worms constitutively release small RNAs *in vitro*, we sequenced small RNAs from replicated (*n* = 5) ES products of *L*. *sigmodontis* gAFs at early (0-24h) and late (48-72h) time points post-harvest. In parallel we tested worm viability over this period of time, showing no significant changes (**Fig 4A**). The depth of sequencing coverage was similar across both time points and we observed a ~2-fold decrease in the number of *L*. *sigmodontis*-specific reads over time when comparing the ES products at 48-72h versus 0-24h (**S6A Table and S4 Fig**). The proportions of the *L*. *sigmodontis*-specific RNA biotypes detected remained the same over this time course. Differential expression analysis identified only one specific difference in the miRNA populations released from 0–24 versus 48–72 hours, miR-34a (**S6B Table and S4 Fig**). However, we note that the actual number of miRNAs that were sequenced in each replicate is low, making statistical conclusions on miRNA-specific changes over time challenging in this model. Together, these results suggest that gAF worms release sRNAs *in vitro*, and the release of sRNAs decays in a time-dependent manner without noticeable changes in worm viability, as previously documented for *B*. *malayi* [8].

It is generally assumed that any extracellular miRNAs detected in ES are stabilized from degradation through encapsulation in EVs or through association with proteins [58]. Consistent with this, RNase sensitivity experiments with pooled ES of gAF demonstrates that the miRNAs are protected from degradation but become susceptible in the presence of detergent (which breaks EVs) or proteinase K treatment (which degrades proteins), **Fig 4B**. To determine whether EVs are present in the ES we used NTA and identified particles ~100 nm in diameter (87.4 nm ± 17.7 nm; mean ± SD) (**Fig 4C**). These were present at relatively low levels compared to those in the ES products of *H*. *bakeri* (~10^7^ particles in *L*. *sigmodontis* gAF ES products vs. ~10^9^ particles in *H*. *bakeri* ES products when using similar volumes of material) [31] and analysis by TEM did not show reveal consistent vesicle-like structures. Attempts to purify the EVs from the ES of gAFs by ultracentrifugation led to a loss of signal by NTA. We can conclude therefore that the extracellular miRNAs are stabilized against degradation but cannot definitively determine at this stage whether stabilization is due to vesicle encapsulation or protein binding, or both.

### Tissue detection of the miRNAs differentially expressed in the ES of gravid adult female worms

**Fig 5:**
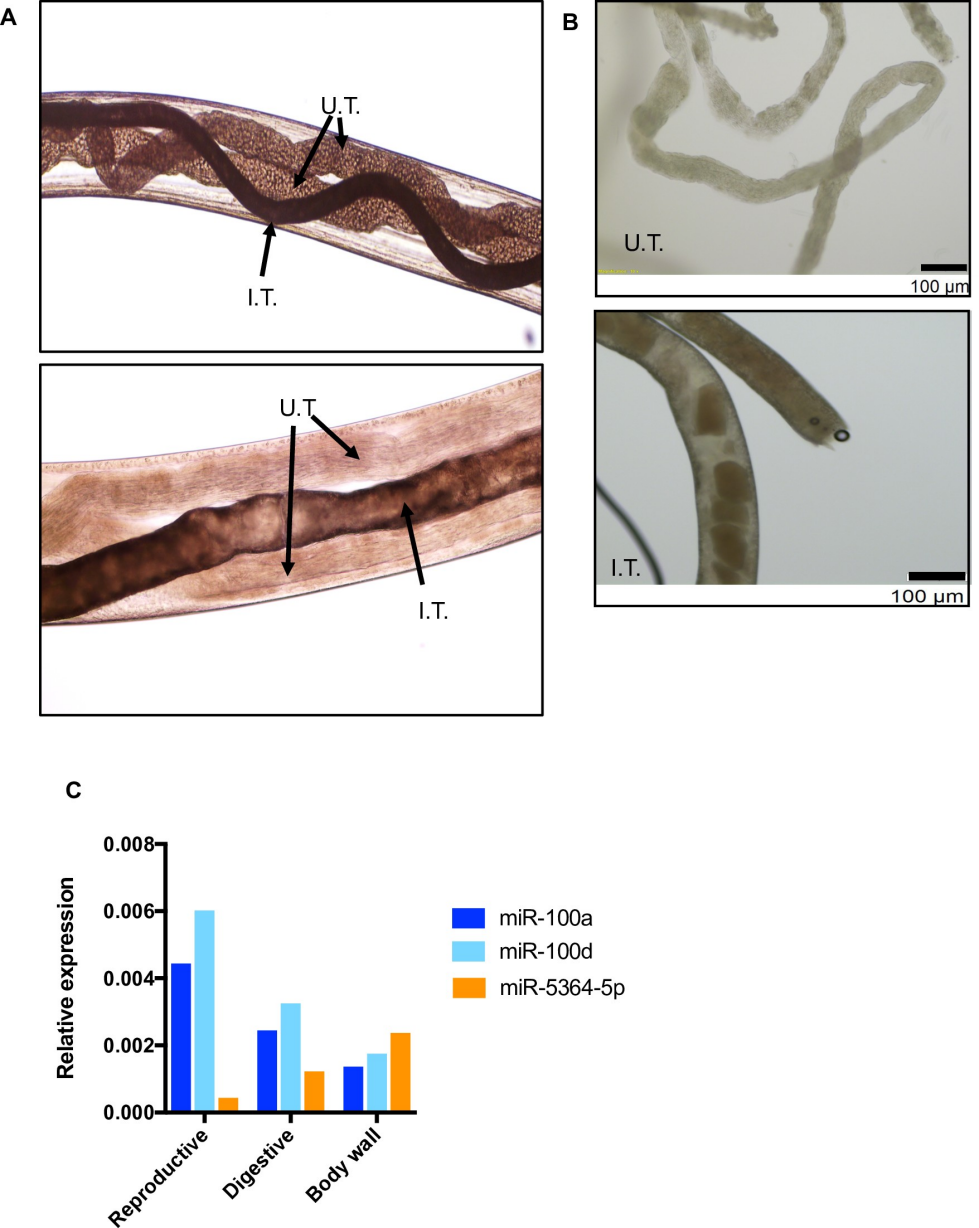
Tissue detection of miRNAs in gravid adult female worms. Adult female worms were dissected and tissues recovered as described in the materials and methods section. Micrographs in (**A**) show a middle body section (upper panel) and anterior section (lower panel), where both uteri (U.T) and the intestinal tube (I.T.) are indicated with arrows, and are anatomically consistent with previously published structures for filarial gravid adult female worms [58,59]. **B**) After dissection, the reproductive tissue (upper panel) and digestive tissue (lower panel) were harvested together with the cuticle. **C)** qRT-PCR of miRNAs shown to be enriched in the ES of gAFs, normalized to the signal of 18S rRNA. The qRT-PCR results described here correspond to a single pooled samples from 60 individual female worms.

To gain insight into the potential origin of the sRNAs detected *in vitro*, we dissected tissues from adult gravid females (e.g. body wall, digestive, and reproductive tissue) (**Fig 5A and 5B**). We observed that both miR-100a-5p and miR-100d-5p (which are enriched in the ES products of gAF worms) are highly abundant in the reproductive tissue but expressed at lower levels in the digestive tissue and the body wall (**Fig 5C**). In contrast miR-5364-5p, which is also enriched in the ES of gAF, is mostly detected in the body wall, with lower expression also detected in the digestive and reproductive tissue (**Fig 5C**) These data suggest that the miRNAs enriched in the ES products of gAF worms are expressed in multiple tissues with secretory capacity. Further work is required to validate the enrichment of miRNAs in specific tissues and to determine whether there is one or multiple origins of the extracellular miRNAs.

### *L*. *sigmodontis*-derived sRNAs are found in host serum and cells during chronic infection

**Fig 6:**
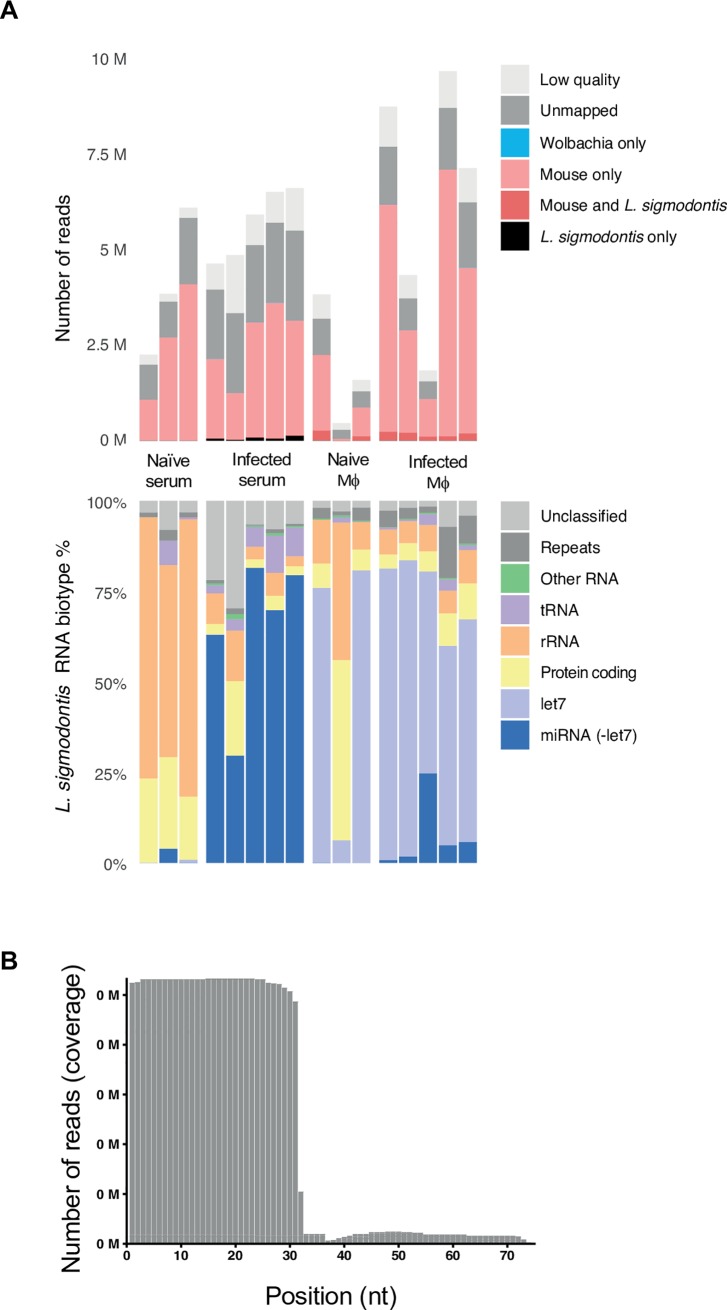
Detection of *L*. *sigmodontis* sRNAs in serum and macrophages from infected jirds by sequencing. **A**) **Top panel**: Composition of the sRNA sequencing libraries from naïve and infected sera and macrophages. The legend qualifies reads that are too short or reads with no adapters (light gray), reads that do not map to any of the genomes used as references in this study (dark gray), as well as reads mapping unambiguously to either the *M*. *musculus* genome (light pink), the *L*. *sigmodontis Wolbachia* (*wLsig)* endosymbiont genome (blue), or the *L*. *sigmodontis* genome (black); we also note the proportion of reads that map to both *M*. *musculus* and *L*. *sigmodontis* (dark pink). **Lower panel**: RNA biotype distribution of the reads that map unambiguously to *L*. *sigmodontis*. Samples were harvested from *L*. *sigmodontis*-infected jirds at day 90 post-infection (*n* = 5). Age- and sex-matched naïve jirds were also included in this study as controls (*n* = 3). The miRNA let-7 is denoted separately from other miRNAs in these plots because of its dominance in the host cell libraries. **B**) Length distribution of reads mapping to *L*. *sigmodontis* tRNAs in these libraries.

**Fig 7:**
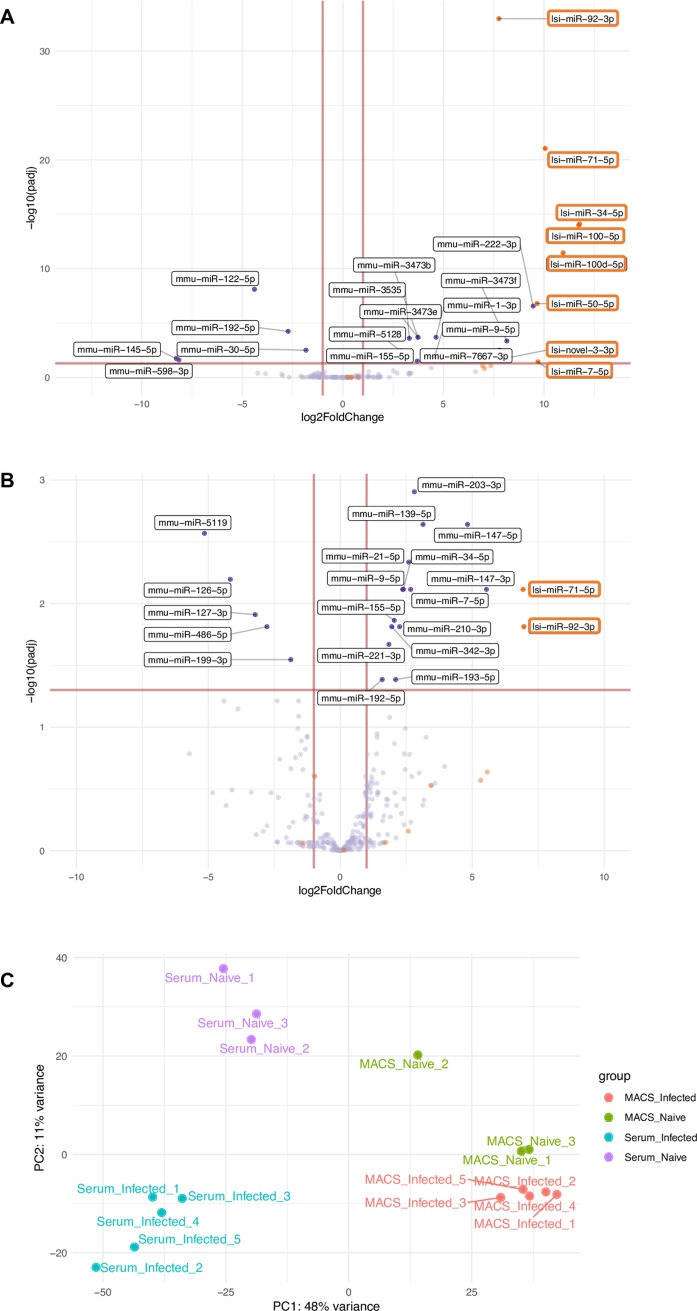
Volcano plots showing differentially expressed miRNAs in serum and macrophages of infected jirds. Scatter plot of *L*. *sigmodontis*-derived miRNAs (orange dots, orange boxes) and murine-derived miRNAs (blue dots, black boxes) that are differentially expressed (DE) in naïve and infected serum (**A**) and macrophages (**B**) from jirds. Horizontal lines represent the cut-off for determining significant DE miRNAs in these datasets (*p* < 0.05). **C**) Principal Component Analysis of the DESeq2 analysis of naïve vs. infected jirds serum and macrophages. The samples are color-coded and the legend on the right indicates the corresponding experimental groups.

To determine the parasite sRNA that can be detected *in vivo* we used Mongolian jirds (*M*. *unguiculatus*), which can accommodate high parasitic burdens, exhibit a much higher microfilaremia [58], and support chronic infection with *L*. *sigmodontis*. At 90 days post infection samples were collected from serum, where microfilariae reside [58], and of macrophages from the pleural/peritoneal exudates. These cells were expected to be the best candidates for detecting parasite sRNA because the adults reside in the pleural/peritoneal cavities [20] and macrophages play a central role during helminth infections, including filarial infections [60–62]. As shown in **Fig 6A** and documented in **S7 Table** we find that most of the *L*. *sigmondontis* reads in infected serum and cells are miRNAs. This profile contrasts with the sRNAs identified in the ES products, where rRNA and tRNA classes dominate (**Fig 1**). We note that the majority of tRNA sequences identified in these samples map to the 5’ end, as observed for the ES products (**Fig 6B**). We observed the presence of 35 miRNAs with > 2 reads in serum or cells from infected jirds (**Table 4, and S8 Table**). Some of the most abundant miRNAs detected in infected samples but not in naïve controls are miR-92-3p, miR-100a-5p and miR-34-5p, which are detected in more than 10,000 reads per million (RPM) of all *L*. *sigmodontis* miRNA reads, whereas other miRNAs were comparatively less abundant (**Table 4, S8 Table)**. A challenge in detecting *L*. *sigmodontis* miRNAs in the *in vivo* environment is the dominance of host sequences, some of which are identical between *L*. *sigmodontis* and M. *unguiculatus*. For example, let-7 is perfectly conserved between these organisms (from nucleotides 1–22) and one of the dominant miRNAs in both naïve and infected host cells, but not serum. The proportion of *L*. *sigmodonotis*-specific reads relate to sequences that are >/ = 23 nt in length and map to the *L*. *sigmodontis* genome based on the presence of an “A” at position 23 (however we note this could also be the host let-7 species with a non-templated addition of “A”); these are denoted in Fig 6A, bottom panel). By comparing infected samples to naïve (which is not possible with ES products) we also note that some *L*. *sigmondontis* sRNAs are identified in naïve serum samples (rRNA and exon-derived RNAs and some miRNAs). These may represent sequences that map to *L*. *sigmodontis* but are also similar to the host or to other potential *in vitro* contaminants (bacteria, fungi). To have confidence that the *L*. *sigmondontis* miRNA reads detected in serum and macrophages represent a *bona fide* signal of infection, we used differential expression of sequences identified in infected versus naïve animals. Through this method we ask which sequences are more abundant in infected samples compared to uninfected, analyzing in parallel miRNAs that are host-derived, *L*. *sigmodontis*-derived or ambiguous (e.g. Let-7). We find eight parasite miRNAs that are reliably detected in serum and these include 5 that are common to the ES of AM, gAF, and mf, and 2 (miR-100a-5p and miR-100d-5p) that are enriched in ES from gAF worms, (**Fig 7A, Table 4, S8 Table**). Differential expression analysis also shows significant detection of miR-92-3p and miR-71-5p in infected macrophages (**Fig 7B**) compared to naïve cells, and identifies several host miRNAs that are up-regulated by infection, contributing to a clustering of infected macrophages by Principal Component Analysis (**Fig 7C and S8 Table**). We also detect two tRNA fragments from tRNA Gly-GCC (Log2FC = 7.43 and *padj* = 1.72E-6) and 5’-tRF-Asp-GTC (Log2FC = 9.11, *padj* = 0.03) to be differentially expressed in serum of infected animals using DESeq2. These two 5’-tRFs display a different sequence than those in the vertebrate host(s) (**S2B** and **S2C Fig**).

**Table 4:** Differentially abundant parasite-derived miRNAs in serum and macrophages from naïve vs infected jirds.

miRNA	Mature miRNA sequence	DE in ES product?	Log2 FC	Adj *p* value (<0.05)
**Enriched in infected serum**				
lsi-miR-34-5p	uggcagugugguuagcugguugu	Common to gAF, AM, mf	11.76	7.91E-15
lsi-miR-100a-5p	aacccguaguuucgaacaugugu	gAF	11.72	1.03E-14
lsi-miR-100d-5p	uacccguagcuccgaauaugugu	gAF	10.94	3.58E-12
lsi-miR-71-5p	ugaaagacauggguagugagacg	Common to gAF, AM, mf	10.05	8.96E-22
lsi-miR-7-5p	uggaagacuugugauuuuguuguuu	Common to gAF, AM, mf	9.69	0.036
lsi-miR-50-5p	ugauaugucugauauucuuggguu	Common to gAF, AM, mf	9.65	1.60E-07
lsi-novel-3-3p	gcccuguuccucucucuucuacc	Common to gAF, AM, mf	8.16	0.015
lsi-miR-92-3p	uauugcacucgucccggccuga	Common to gAF, AM, mf	7.75	1.08E-33
**Enriched in infected Macrophages**				
lsi-miR-92-3p	uauugcacucgucccggccuga	Common to gAF, AM, mf	6.95	0.015
lsi-miR-71-5p	ugaaagacauggguagugagacg	Common to gAF, AM, mf	6.92	0.0076

## Discussion

The discovery that RNA is released by parasitic nematodes opens many avenues for further investigation into their functional properties and diagnostic utility. In this report, we used the rodent filarial model *L*. *sigmodontis* to address some foundational questions of relevance to the field: which parasite extracellular sRNAs are most abundant *in vitro*, how does this compare *in vivo*, and can we assign the origin of the sRNAs that we detect in host fluids to a specific parasite lifecycle stage? Our results demonstrate that all larval and adult stages of *L*. *sigmodontis* secrete sRNAs *in vitro* (~20–60 nt in length) and the small species (<40 nt) are dominated by rRNA and tRNA fragments as well as sRNAs that map to exonic regions in the *L*. *sigmondontis* genome. We do not yet know if these are degradation products or specific fragments whose release is controlled. We also identify miRNAs in ES from all lifecycle stages and these are consistently much less abundant than the other sRNA categories *in vitro*. We show that most of the miRNA species in ES from one lifecycle stage are common to the ES of all lifecycle stages. We note that the quantity and diversity of miRNAs detected in the ES of gAF is larger than the other lifecycle stages, consistent with previous proteomic characterization of the ES products from *L*. *sigmodontis* [7]. We infer that adult female worms prolifically release both proteins and sRNAs *in vitro*, perhaps as a component of the uterine fluids (released during mf birth), coupling, defecation, or other physiological processes [63–66]. A key question in this field is whether any of the miRNAs detected *in vivo* are derived from gravid adult females, the target of many diagnostic strategies [17]. Our *in vitro* data identify several miRNAs enriched in the ES of gAF that are also detected *in vivo* and found in the reproductive tissue of the worms (e.g. members of the miR-10/miR-100 family). A hypothesis is that miR-100a-5p and miR-100d-5p which are detected in the serum of infected animals could derive from the gAF. However, these miRNAs are detected at some level in the ES from all stages, and we cannot exclude the potential (and likely) contribution of miRNAs secreted by mf to the pool of circulating parasite-derived miRNAs, as proposed previously [12]. It is of interest that in *Brugia*, miR-100a and miR-100d are within the same gene, Bm1_19500, that encodes for the recombinant factor GdRad54, which is involved in DNA repair and recombination events [37,67], likely to take place during embryogenesis. Further work is required to understand whether the gAF-enriched miRNAs reach the bloodstream prior to infection patency.

Based on the literature to date, it is assumed that miRNAs released from parasitic nematodes are stabilized through encapsulation in EVs, and we have found specific sRNAs stabilized by EVs in Clade V gastrointestinal nematodes [13,31]. However, in mammalian systems, non-vesicular extracellular miRNAs can be stabilized by proteins such as Argonautes and high density lipoproteins [68–71]. From our analyses here, we conclude that the majority of filarial miRNAs released by the gravid adult female worms are protected from degradation by RNases but we do not yet know if this exclusively involves EVs or other non-vesicular complexes. We identify relatively low levels of EVs in the ES from gAFs in *L*. *sigmondontis* compared to the ES from the Clade V gastrointestinal nematode *H*. *bakeri* [31]. We also do not observe a clear signal for secondary siRNAs in the ES of *L*. *sigmondontis*, in contrast to the EVs of Clade V nematodes. Collectively, these studies suggest diversity in the composition of extracellular RNAs among different parasitic nematodes and it will be of interest to understand their functional properties in relation to different niches in the host.

We show that during the infection *in vivo*, *L*. *sigmodontis* miRNAs can indeed be detected in macrophages of the pleural cavity, at low but reproducible levels, thus opening the possibility that they play a functional role in parasite-host communication. Previous studies have shown internalization of EVs derived from nematode parasites by macrophages *in vitro* [8,72,73] and our work adds to a recent report demonstrating internalization of parasite miRNAs in host cells *in vivo* [74]. Another recent report showed the detection of miRNAs from *H*. *contortus* in the lymph nodes of infected animals by qRT-PCR, suggesting these can be detected at sites distant to the parasite [75]. It will be of interest to understand the mechanism of parasite miRNA internalization *in vivo* and whether this involves EVs or protein complexes [76]. It is also of interest to understand why parasite miRNAs are a more dominant category of parasite-derived sequences *in vivo* compared to *in vitro* (where their levels are low in the ES compared to rRNAs and tRNAs). This could suggest that release of sRNAs is sensitive to environmental conditions (such that some of the *in vitro* observations may not be relevant *in vivo*) or that the stability of the sRNAs once released is impacted by the *in vivo* context (for example if the miRNAs associate with host proteins but the rRNAs do not, this could lead to different half-lives *in vivo*). It also remains possible that other cell types *in vivo* internalize parasite sRNAs which we have not examined. A limitation of our study is that we cannot yet distinguish between these possibilities. A further limitation of our study is that, apart from miRNAs and tRNA sequences, we have analyzed the other RNA classes as a group. It remains possible that individual sequences within the categories could show enrichment in certain contexts and this requires further study. Despite these limitations, the analysis of miRNAs lends confidence to the relevance of parasite sRNAs *in vivo*. The signal for parasite miRNAs *in vivo* seems unlikely to result from a mis-assignment of host miRNA reads to the parasite, since miR-71-5p is distinct in sequence from any host miRNA. Furthermore, miR-92-3p is similar in the first 20 nt between host (gerbil) and *L*. *sigmondontis* but the nucleotides >20 nt in length map to the *L*. *sigmondontis* sequence and not to host (**S3C Fig**). Importantly, the parasite-mapping miR-92 reads are more abundant in infected macrophages compared to naïve cells (**Fig 7B**). Further work is required to understand the host or parasite co-factors with which parasite miRNAs associate in order to test their functional properties in cells.

## Supporting information

S1 Table List of qPCR primers used in this study. (PDF)

S2 Table Genome mapping and diversity of RNA biotypes detected in *L. sigmodontis* adult worms and ES products. This table contains two tabs. **S2A Table**: Genome mapping and diversity of RNA biotypes detected in *L*. *sigmodontis* adult worms and ES products. The ES samples in the table are labelled with numbers denoting replicates according to the following naming: **vL3s** = vector-derived L3s; **hL3** = host-derived L3s; **L4** = 4th larval stage; **AM** = Adult Male; **gAF** = gravid Adult Female; **mf** = microfilariae. **S2B Table**: miRNA identification of reads mapping perfectly to both *M*. *musculus* and *L*. *sigmodontis* using miRDeep2 (score >1).
(XLSX)

S3 Table tRNAScan-SE results. In addition to the overall statistics obtained from tRNAScan-SE, that includes locus position and coordinates as well as potential introns within each of the tRNAs identified (45), this table also contains the predicted RNA sequences in 5’-3’ orientation. Note that the column labelled as “tRNA #” indicates the number of different tRNAs identified in the same genomic scaffold.
(XLSX)

S4 Table Top 15 of the most abundant 5’-derived tRFs detected in the ES products of larval and adult stages of *L. sigmodontis*. This table reports the tRNAs identified in the ES products from *L*. *sigmodontis* adult males (AM; *n* = 10), gravid adult females (gAF; *n* = 12), and microfilariae (mf; *n* = 8), as well as the specific anticodon and genomic loci in the *L*. *sigmodontis* draft genome. Additionally, the table include the type of tRNA identified and its cognate anticodon, the corresponding tRNA sequence, the total tRNA read counts across all sequencing libraries, the normalized reads per million, and the average number of reads per lifecycle stage tested.
(XLSX)

S5 Table Prediction of known and novel *L. sigmodontis* miRNAs in ES products obtained in vitro. This table is composed of two tabs; **S5A Table**: predicted known and novel miRNAs from libraries prepared from (untreated or 5’PPase-treated) total worm lysates (gravid adult females and adult males) or untreated vs. 5’PPase-treated ES products from gAF worms (ES). Note that the 5’PPase-treated libraries are denoted with the letters “PP”. Samples ES046 and ES053 correspond to ES products from gAF worms that were either untreated or 5’PPase-treated (“ES046PP” or “ES053PP”). Other samples included in this table include the ES products from vector-derived L3s (vL3s), host-derived L3s (hL3s), L4s, juvenile adult males (jAM), pre-gravid adult females (pgAF), gravid adult females (gAF), adult males (AM), and microfilariae (mf). **S5B Table**: Rational for assigning miRNAs ID to the products obtained from miRDeep2, as explained in materials and methods.
(XLSX)

S6 Table Prediction of known and novel *L. sigmodontis* miRNAs in ES products obtained at early (0-24h; *n* = 5) and late (48–72h; *n* = 5) time points *in vitro*. This table is composed of three tabs; **S6A Table**: Diversity of RNA biotypes detected in *L*. *sigmodontis in vitro* gravid adult female (gAF) ES products over time. This tab also contains information of the proportion of reads unambiguously mapping to either *M*. *musculus* or *wLsig*. **S6B Table**: predicted known and novel miRNAs from libraries prepared from gAF ES products at early (0-24h) and late (48-72h) time points using miRDeep2. S6C Table: Differential expression analysis of *L*. *sigmodontis* miRNAs at 0-24h versus 48–72 h post harvest.
(XLSX)

S7 Table RNA biotype diversity in serum and macrophage cells from naïve (*n* = 3) and *L. sigmodontis*-infected jirds (*n* = 5). This table includes an overview of the total read counts obtained for each library, as well as the high-quality reads obtained after adaptor trimming and filtering. Additionally, we also report the proportion of reads mapping to either *M*. *musculus* or *wLsig*. Given the sequence conservation of Let-7 between *L*. *sigmodontis* and *M*. *musculus*, we report the *L*. *sigmodontis*-specific read counts after subtracting those derived from let-7.
(XLSX)

S8 Table miRNAs detected in serum from naïve and infected jirds. This table contains three tabs; **S8A Table**: Total counts for mapping specifically to either the murine genome (“mmu”) or the *L*. *sigmodontis* genome (“lsi”) in both naïve (*n* = 3) and infected (*n* = 5) serum and macrophages. These counts were used as inputs for DESeq2 analysis as detailed in materials and methods. **S8B Table**: DESeq2 results from serum analysis. Those miRNAs with an adjusted p value *(padj)*<0.05 are shown in bold. **S8C Table**: DESeq2 results from macrophages analysis. Those miRNAs with a *padj* <0.05 are shown in bold.
(XLSX)

S1 Fig Small RNA profile of the ES products of larval and adult stages of *L. sigmodontis*. Total RNA (1 μL) purified from *in vitro L*. *sigmodontis* ES products from the noted lifecycle stages was loaded into a Bioanalyzer small RNA chip. Representative electropherograms are shown indicating two clear populations of RNAs in ES products with different lengths: 20–30 nt and 40–60 nt. The concentrations of RNA measured by the Bioanalyzer are noted below each lane.
(PDF)

S2 Fig 5’-derived tRNA halves are abundantly detected in *L. sigmodontis* ES products. **A)** Length distribution of reads mapping to putative *L*. *sigmodontis* tRNAs in ES products from adult males (AM), gravid adult females (gAF), and microfilariae (mf), in three independent replicates (denoted as Rep1, Rep2, or Rep3). Primary structure of tRNA-Gly-GCC (**B**) and tRNA-Asp-GTC (**C**) consistently found in serum from naïve and infected jirds.
(PDF)

S3 Fig Conservation of extracellular miR-10 family and miR-92, and predicted top 3 novel miRNAs detected in ES products from larval and adult stages of *L. sigmodontis*. **A**) primary structure of the filarial miR-10 family sequences reported in *L*. *sigmodontis* (lsi), *D*. *immitis* (dim), *L*. *loa* (llo), and *O*. *ochengi* (ooc), and compared to the miR-10 family sequence in *H*. *sapiens* (hsa). **B**) as in (A), showing the primary structure of the miR-92 family detected in *L*. *sigmodontis* (lsi) and the corresponding sequences in *H*. *sapiens* (hsa) and *M*. *musculus* (mmu). **C**) Secondary structures of the most abundant novel miRNAs (>50% of total read counts for novel miRNAs) predicted by miRDeep2 using the Vienna RNAfold package. Minimum free energy values (reported as kcal/mol) where estimated using the RNAfold webserver (http://rna.tbi.univie.ac.at/cgi-bin/RNAWebSuite/RNAfold.cgi).
(PDF)

S4 Fig Genome mapping and diversity of RNA biotypes detected in *L. sigmodontis* ES products at early (0-24h) and late (48-72h) time points. **Top panel**: legend qualifies reads that are too short or reads with no adapters (light gray), reads that do not map to any of the genomes used as references in this study (dark gray), as well as reads mapping unambiguously to either the *M*. *musculus* genome (light pink), the *L*. *sigmodontis Wolbachia* (*wLsig)* endosymbiont genome (blue), or the *L*. *sigmodontis* genome (black); we also note the proportion of reads that map to both *M*. *musculus* and *L*. *sigmodontis* (dark pink). **Lower panel**: RNA biotype distribution of the reads that map unambiguously to *L*. *sigmodontis*.
(PDF)
