# Supplementary material for: Comparative analysis of small RNAs released by the filarial nematode Litomosoides sigmodontis in vitro and in vivo
Source: PLoS Negl Trop Dis. 2019 Nov 26;13(11):e0007811. doi: 10.1371/journal.pntd.0007811 (PMC6903752; doi:10.1371/journal.pntd.0007811)
Supplement: S1 Table — (PDF) [file pntd.0007811.s001.pdf]

S1 Table. List of qPCR primers used in this study

Target	DNA sequence (5'-3')	Company	Slope	Amplification factor	Efficiency (E)	Reference
**RT1**	ACTGTACGTCTGCCATTAGCTT	IDT	-3.576	1.0	90.4%	This study
**18S rRNA Forward***	GTACAAAGGGCAGGGACGTA	IDT	ND	ND	ND	(47)
**18S rRNA Reverse***	CATTGCCGAAAGGTACTGGT	IDT	ND	ND	ND	(47)
**miR-71-5p**	TGAAAGACATGGGTAGTGAGAC	Invitrogen	-4.093	1.76	75.5%	This study
**Lin-4-5p**	TCCCTGAGACCTCTGCTGCGA	Invitrogen	-3.987	1.78	78.2%	This study
**miR-100a-5p**	AACCCGTAGTTTCGAACATGTGT	Invitrogen	-3.051	2.13	112.7%	This study
**miR-100d-5p**	TACCCGTAGCTCCGAATATGT	Invitrogen	-3.205	2.05	105.1%	This study
**miR-5364-3p**	CGAGGTATTGTTTATTGGCTGA	Invitrogen	-3.703	1.86	86.2%	This study

*Sequences correspond to the 18S rRNA of *Litomosoides sigmodontis.* ND = not determined
