# Supplementary figures and images for: Comparative analysis of small RNAs released by the filarial nematode Litomosoides sigmodontis in vitro and in vivo
Source: PLoS Negl Trop Dis. 2019 Nov 26;13(11):e0007811. doi: 10.1371/journal.pntd.0007811 (PMC6903752; doi:10.1371/journal.pntd.0007811)

S1 Fig.

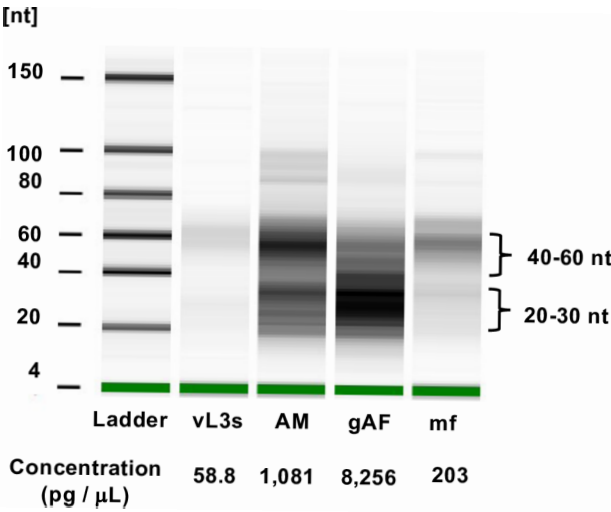

Supplement: S1 Fig — Total RNA (1 μL) purified from in vitro L. sigmodontis ES products from the noted lifecycle stages was loaded into a Bioanalyzer small RNA chip. Representative electropherograms are shown indicating two clear populations of RNAs in ES products with different lengths: 20–30 nt and 40–60 nt. The concentrations of RNA measured by the Bioanalyzer are noted below each lane. (PDF) [file pntd.0007811.s009.pdf]

## S2 Fig.

**A**

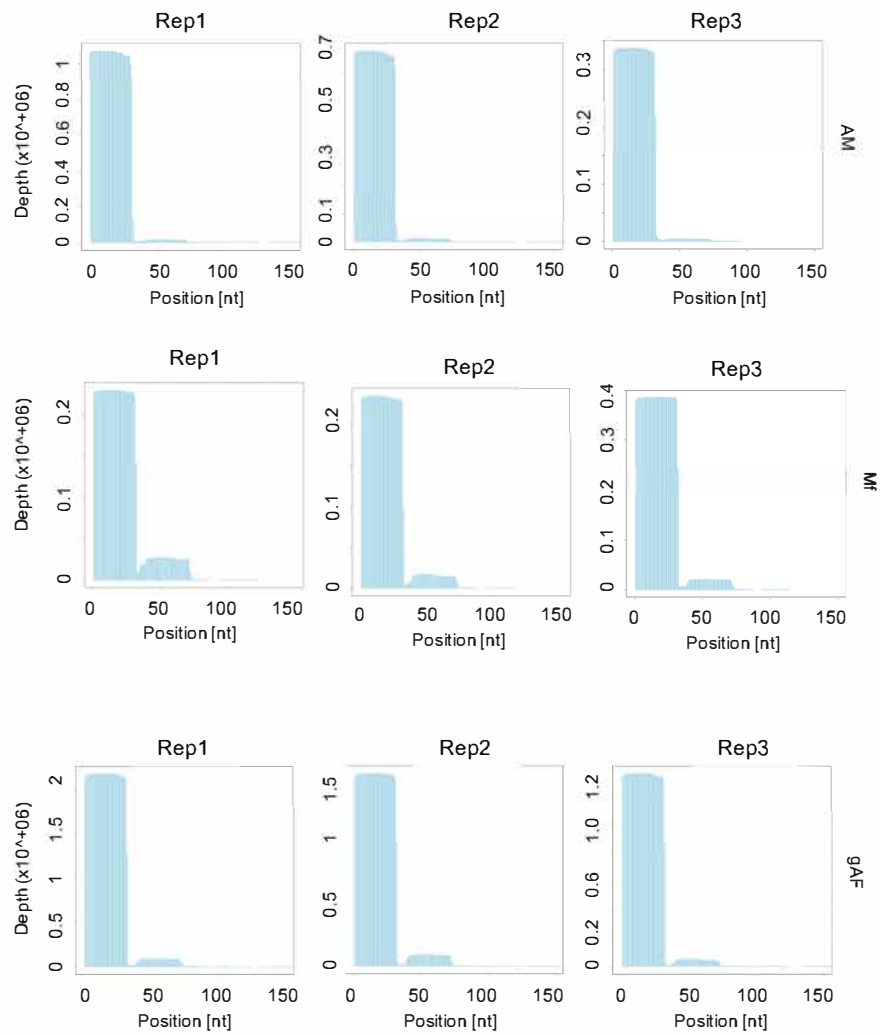

**B**

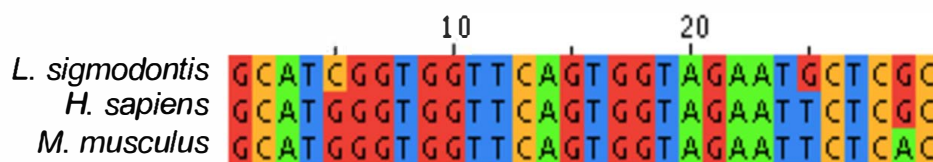

**C**

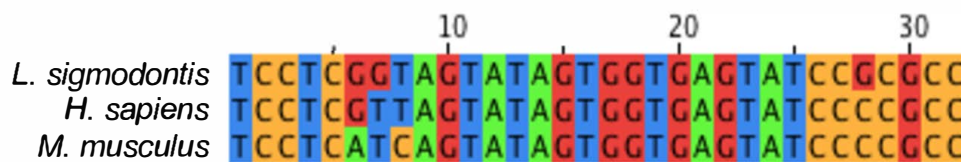

Supplement: S2 Fig — A) Length distribution of reads mapping to putative L. sigmodontis tRNAs in ES products from adult males (AM), gravid adult females (gAF), and microfilariae (mf), in three independent replicates (denoted as Rep1, Rep2, or Rep3). Primary structure of tRNA-Gly-GCC (B) and tRNA-Asp-GTC (C) consistently found in serum from naïve and infected jirds. (PDF) [file pntd.0007811.s010.pdf]

S3 Fig.

A

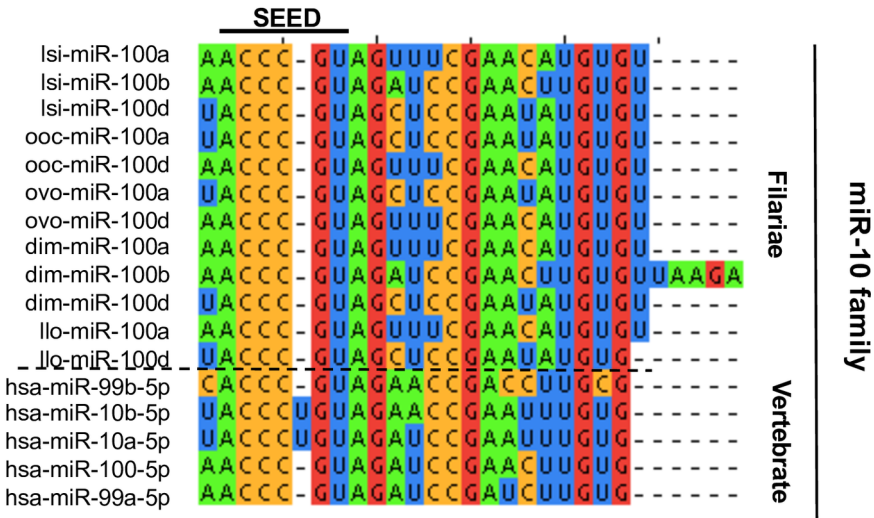

B

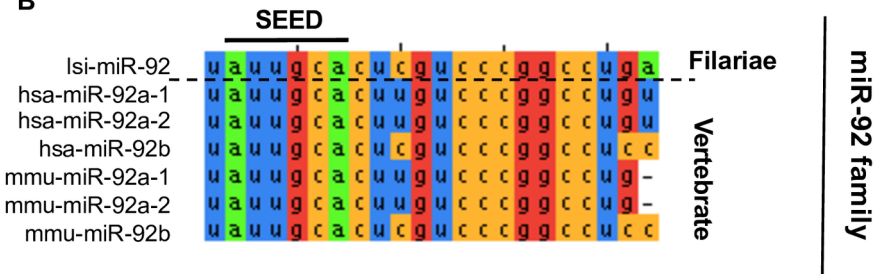

C

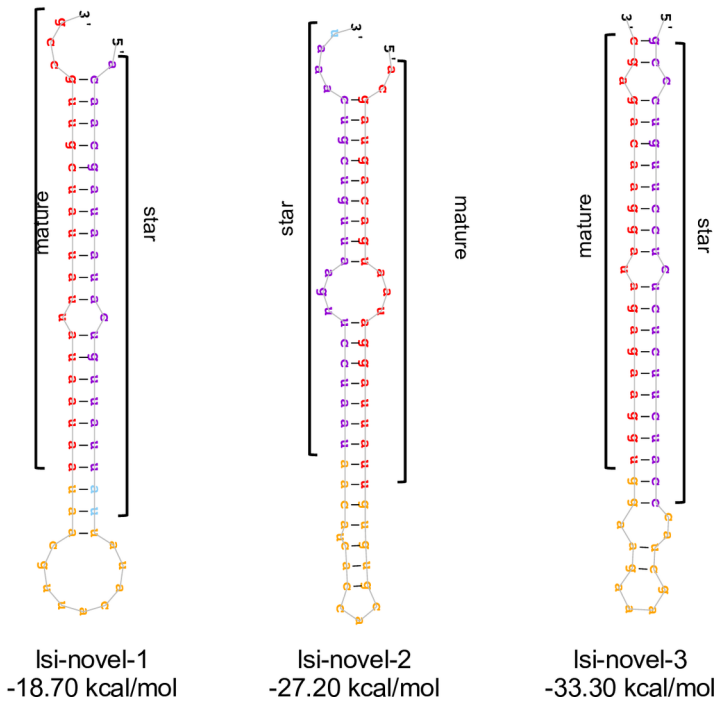

Supplement: S3 Fig — A) primary structure of the filarial miR-10 family sequences reported in L. sigmodontis (lsi), D. immitis (dim), L. loa (llo), and O. ochengi (ooc), and compared to the miR-10 family sequence in H. sapiens (hsa). B) as in (A), showing the primary structure of the miR-92 family detected in L. sigmodontis (lsi) and the corresponding sequences in H. sapiens (hsa) and M. musculus (mmu). C) Secondary structures of the most abundant novel miRNAs (>50% of total read counts for novel miRNAs) predicted by miRDeep2 using the Vienna RNAfold package. Minimum free energy values (reported as kcal/mol) where estimated using the RNAfold webserver (http://rna.tbi.univie.ac.at/cgi-bin/RNAWebSuite/RNAfold.cgi). (PDF) [file pntd.0007811.s011.pdf]

S4 Fig.

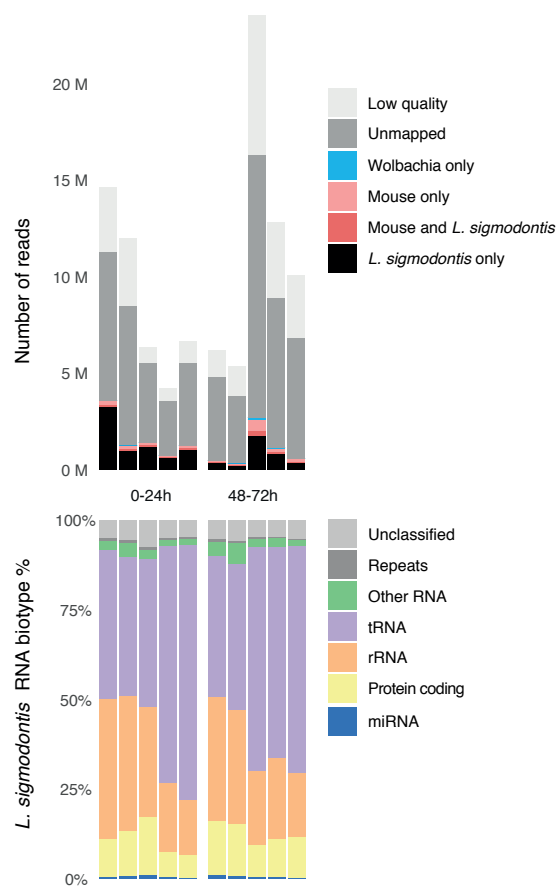

Supplement: S4 Fig — Top panel: legend qualifies reads that are too short or reads with no adapters (light gray), reads that do not map to any of the genomes used as references in this study (dark gray), as well as reads mapping unambiguously to either the M. musculus genome (light pink), the L. sigmodontis Wolbachia (wLsig) endosymbiont genome (blue), or the L. sigmodontis genome (black); we also note the proportion of reads that map to both M. musculus and L. sigmodontis (dark pink). Lower panel: RNA biotype distribution of the reads that map unambiguously to L. sigmodontis. (PDF) [file pntd.0007811.s012.pdf]
